# A Multi-Omics Framework Reveals Tumor Heterogeneity and Predicts Therapeutic Targets in Renal Cell Carcinoma

**DOI:** 10.3390/ijms27104456

**Published:** 2026-05-15

**Authors:** Xiangzhe Yin, Zihe Zhou, Yunzhu Xue, Yangxinyue Zheng, Wentong Yu, Zhichao Geng, Yanwu Sun, Lu Wang, Zushun Chen, Siyao Wang, Li Wang, Hongying Zhao

**Affiliations:** College of Bioinformatics Science and Technology, Harbin Medical University, Harbin 150081, China

**Keywords:** multi-omics analysis, spatial transcriptomics, metabolic reprogramming, renal cell carcinoma, gene regulatory networks

## Abstract

Tumor cell heterogeneity and multicellular interactions critically influence drug resistance, recurrence, and prognosis. Here, CPcellsubpopulation, a computational framework integrating scRNA-seq, bulk RNA-seq, and clinical data was developed to identify cancer progression-associated cell subpopulations. Then, the integrated analyses of scRNA-seq and spatial transcriptomics were performed to predict potential interactions, identify critical transcription factors, and predict candidate anticancer drugs. Across nine cancers, we detected cancer progression-associated cell subpopulations significantly linked to prognosis, with consistent patterns across cancer types. In renal cell carcinoma (RCC), we identified conserved metabolic^high^ *UBE2C+* cancer cells linked to poor outcomes, metabolic reprogramming and low differentiation, and *PLK1+* NK cells, plasma cells, and *CDC20*+ macrophages associated with advanced stages and unfavorable prognosis. Spatial mapping revealed spatial association of RCC progression-associated cancer and immune cell subpopulations, suggesting the potential role of the *VEGF*, *GDF*, *PTN* and *IL16* pathways in the remodeling of the tumor microenvironment. Gene regulatory network analysis highlighted *RAD21* as a key regulator linking metabolism and therapy resistance. This study provides a systematic pipeline to delineate cancer progression-associated cell subpopulations, uncovers metabolic^high^ *UBE2C+* cancer cells as progression-associated tumor cell population, and nominates critical regulators and compounds as therapeutic targets.

## 1. Introduction

Kidney cancer is the seventh most common cancer in the world, and its incidence is on the rise. Renal cell carcinoma (RCC) is the most common form and is a heterogeneous disease [1]. RCC progression involves abnormalities in multiple malignant cell populations and tumor microenvironment (TME), as well as alterations in their molecular communications [2]. The tumor microenvironment of RCC is accompanied by a high level of terminally exhausted *CD8*+ T cells and suppressive M2-like macrophages, which leads to severe immunosuppression and immune resistance [3]. The occurrence and progression of RCC originating from multi-step genetic alterations may lead to abnormal gene expression. *PABPC1L* promotes immune escape and immunosuppression in RCC by upregulating *IDO1*, inducing T-cell dysfunction [4]. Tumor cells promote their growth, progression, and metastasis by releasing soluble factors and recruiting cells, and gradually induce phenotypic changes in TME [5]. However, the phenotypic heterogeneity and location relationships of tumor cells and immune cells in RCC remain elusive.

With the advent of single-cell RNA sequencing (scRNA-seq) and spatial transcriptomics (ST), these technologies have been regarded as unprecedented tools to dissect a comprehensive gene expression atlas and spatial arrangement information [6]. At the single-cell level, fibroblast-derived *FGF7* triggers *AKT* activation, promoting cell growth and invasion of RCC tumor cells [7]. Notably, single-cell dissociation lacks spatial information, which is indispensable for a comprehensive understanding of the pathophysiology and progression of RCC. ScRNA-seq and ST analyses have revealed the metabolic subtypes that contribute to the RCC progression and poor prognosis [8]. Therefore, the integration of ST and scRNA-seq helps to accurately characterize the tumor microenvironment and cell interaction patterns.

To reveal the heterogeneity of the tumor immune microenvironment of RCC, we designed a new computational approach to identify cancer progression-associated cell subpopulations. Next, high-throughput scRNA-seq and ST data were integrated to construct a large-scale single-cell and spatial transcriptomics map of RCC to reflect the heterogeneity of different tumor tissues, tumor cells, and immune cells, as well as the signaling interactions between tumor cells and immune cells. Furthermore, we identified critical transcription factors (TFs) in tumor cells and predicted specific drug candidates for RCC. Together, this work reveals the cancer cells related to the RCC progression and critical TFs, providing a strong theoretical basis for improving the clinical diagnosis and targeted therapy of RCC.

## 2. Results

### 2.1. Identification of Cancer Progression-Associated Cell Subpopulations Across Cancer Types

**Figure 1:**
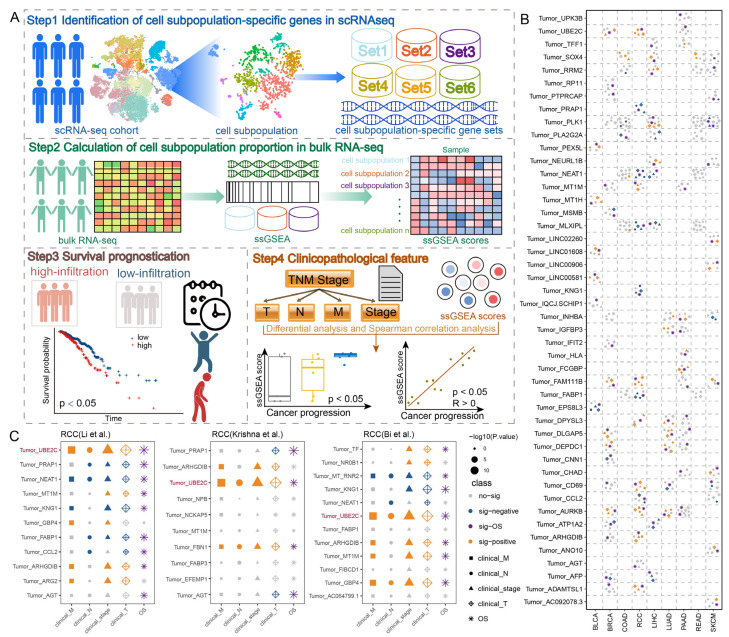
**Schematic overview of the method for identifying cancer progression-associated cell subpopulations**. (**A**) Schematic workflow. (**B**) Distributions of cancer progression-associated cell subpopulations in pan-cancer. (**C**) Distributions of cancer progression-associated cell subpopulations in RCC. Dot shapes in panels (**B**) and (**C**) refer to clinical variables (either T, N, M, overall clinical staging or overall survival), colors refer to significance (either significant positive correlation, significant negative correlation, significant for OS or non-significant), and sizes refer to the significance degree expressed in −log10(*p*-value), according to the legend located on the right-side of the last graph in panel (**C**).

To systematically identify cell subpopulations that may contribute to cancer progression, we designed CPcellsubpopulation, a new computational framework designed to identify cell subpopulations associated with cancer progression (Figure 1A). In the present study, CPcellsubpopulation was applied to systematically identify cancer progression-associated cell subpopulations across nine cancer types, ultimately identifying 46 cancer progression-associated cell subpopulations (Figure 1B). Collectively, 68.1% of the cancer cell subpopulations displayed a significant association with the progression of T stage. For instance, the infiltration level of *FAM111B*+ cancer cells significantly increased with T stage and was significantly correlated with prognosis of breast cancer (BRCA), skin cutaneous melanoma (SKCM), and RCC. Activation of *FAM111B* accelerates the progression of breast cancer [9]. Regarding progression at the overall TNM stage, 46.8% of the cancer cell subpopulations showed a significant difference. *AURKB*+ cancer cells were significantly correlated with prognosis of BRCA, RCC, and lung adenocarcinoma (LUAD), and increased significantly with overall TNM stage. Studies have shown that *AURKB* overexpression promotes tumor cell proliferation and growth in triple-negative breast cancer [10]. Additionally, 12.8% and 21.3% of cancer cell subpopulations were significantly associated with the progression of M stage and N stage, respectively. Taking renal cell carcinoma (RCC) as an example, to elucidate the heterogeneity of the tumor microenvironment and determine RCC progression-associated cell subpopulations, we obtained scRNA-seq data from 10 RCC patients. After stringent quality control measures, a total of 243,864 cells were analyzed across tissues (Figure S1A,C). Graph-based clustering and canonical cell marker annotation revealed eleven major cell types, including B-cells, dendritic cells, endothelial cells, epithelial cells, fibroblasts, macrophages, mast cells, monocytes, NK cells, plasma cells, and T-cells (Figure S1D). Next, to confirm the pivotal cancer cell subpopulations that initiate RCC, we extracted epithelial cells and used Cancer-Finder to identify 12,520 cancer cells [11]. By unsupervised clustering, we identified 11 cancer cell subpopulations. Notably, *UBE2C+* and *NEAT1*+ cancer cells were dysregulated in M, N, T, and overall TNM stages, as well as in prognosis of RCC (Figure 1C). *UBE2C+* cancer cells were significantly associated with M, N, T, overall TNM stage, and overall survival, which is consistent with the results of previous studies [12].

Since RCC has a large number of cancer progression-associated cell subpopulations, we used RCC as an example to verify the effectiveness of the method. Two additional independent RCC scRNA-seq cohorts from Bi et al. and Krishna et al. were obtained, containing 12 RCC patients and 178,088 cells. A total of 16,617 cancer cells were identified. The CPcellsubpopulation method was applied to the validation datasets to identify cancer progression-associated cell subpopulations. In line with the outcomes from the RCC discovery dataset, two validation datasets consistently identified the *UBE2C+* cancer cell subpopulation as a cancer progression-associated cell subpopulation (Figure 1C). The infiltration level of the *UBE2C+* cancer cell subpopulation was consistently found to increase gradually across M, N, T, and overall TNM stages and was significantly correlated with patient prognosis. In conclusion, we developed a new computational approach that effectively identifies cancer progression-associated cell subpopulations and helps identify more precise biomarkers for cancer patients.

### 2.2. Identification of Metabolic^high^ UBE2C+ Cancer Cells as Featured Subpopulations of RCC Progression

**Figure 2:**
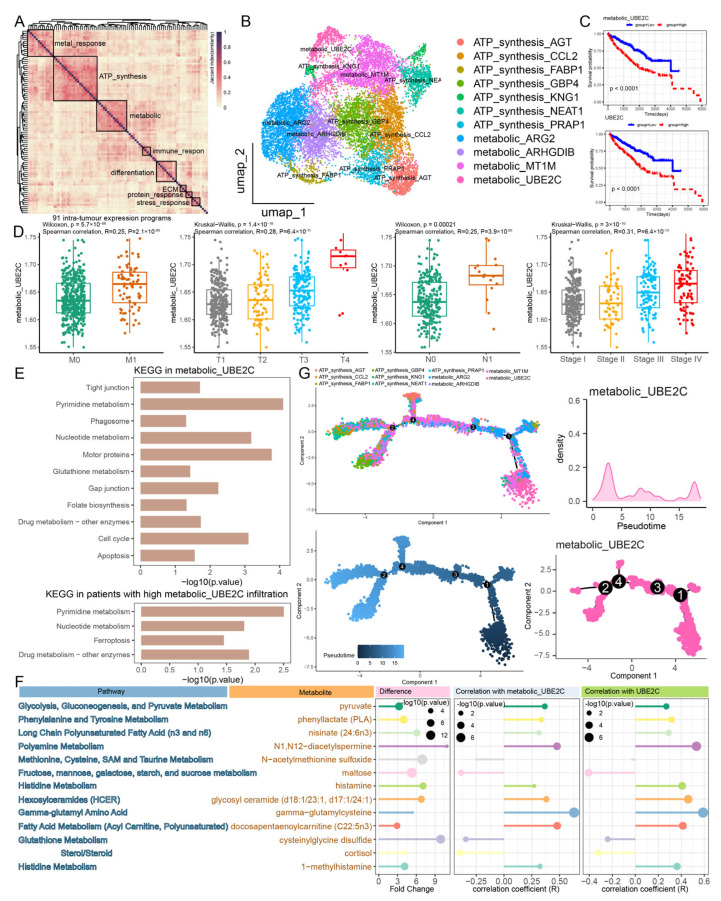
**Identification of metabolic^high^ *UBE2C+* cancer cells as featured subpopulations of RCC tumorigenesis.** (**A**) Heatmap depicting shared expression programs across 22 patients. (**B**) UMAP showing cancer cell subpopulations identified by marker gene expression. (**C**) The Kaplan–Meier survival curves of RCC patients stratified by cancer cell subpopulations infiltration. (**D**) Comparison of absolute infiltration proportion of metabolic^high^ *UBE2C+* cancer cells in M classification, N classification, T classification, stage. (**E**) Pathway enrichment of differentially expressed genes in metabolic^high^ *UBE2C+* cancer cells and patients with high infiltration of metabolic^high^ *UBE2C+* cancer cells. (**F**) Bubble map of metabolite showed the differences in metabolites between cancer samples and normal samples (**left**), correlation between metabolite abundance and infiltration of metabolic^high^ *UBE2C+* cancer cells (**middle**), and correlation between metabolite abundance and *UBE2C* (**right**). −log10(*p*-value) indicates the significance level. (**G**) Pseudotime analysis by Monocle2 shows the potential evolutionary trajectory of cancer cell subpopulations. Dot colors refer to cancer cell subpopulations.

The differences in infiltrating cancer cell subpopulations across clinicopathological features suggest that the remodeling of cancer cells plays functional roles in RCC progression. To explore intratumoral expression heterogeneity and functional characteristics in cancer progression-associated cell subpopulations, we integrated three single-cell datasets of RCC, comprising 22 samples and 29,137 cancer cells. We applied non-negative matrix factorization (NMF) to dissect 91 intratumoral expression programs from 22 RCC patients. We classified eight modules that included highly similar programs across the multiple samples (Figure 2A, Table S1). More specifically, the metal response module consisted of metal ion-related genes (*B2M* and *FTL*) [13]. An ATP synthesis module was characterized by expression of genes such as *MT-CO1* and *MT-CO3*, thus representing energy generation. A metabolic module was enriched for genes such as *ENO1* and *PKM*, which are related to the metabolic process [13]. An immune response module was detected, containing immune-related genes, such as *HLA-C*, *HLA-B*, and *HLA-A* [13]. A differentiation module, consisting of *SPP1*, *ACTB*, and *TMEM176A*, reflected the functions related to cell differentiation [14]. An ECM (extracellular matrix) module was characterized by genes such as *CAV1*, *ACTG1*, and *VCAN*, and was related to focal adhesion and extracellular matrix. Genes such as *FOS* and *JUN* were found in the stress response module, indicating that this module is related to stress response signaling. A protein response module was enriched for genes such as *HSPA1A*, *DNAJB1*, and *HSPA1B*, which are related to protein function [15].

Furthermore, we investigated the associations between each module and *UBE2C+* cancer cells in three single-cell datasets of RCC. It was observed that the metabolic module, which served as a prominent tumor module within the *UBE2C+* cancer cells, was consistent across all three single-cell datasets (Figure 2B, and Figures S2A and S3A). Consequently, we designated the cancer-associated cell subpopulation in RCC as the metabolic^high^ *UBE2C+* cancer cells. To assess the clinical relevance of metabolic^high^ *UBE2C+* cancer cells, we observed that patients with higher cell infiltration of metabolic^high^ *UBE2C+* cancer cell had a significantly worse overall survival (Figure 2C, Table S2). Additionally, metabolic^high^ *UBE2C+* cancer cells from two independent scRNA-seq cohorts exhibited consistent results (Figure S2B). Notably, the proportion of metabolic^high^ *UBE2C+* cancer cells exhibited an increasing trend with the progression of TNM staging and tumor stage (Figure 2D and Figure S3B). In the two independent RCC scRNA-seq cohorts, metabolic^high^ *UBE2C+* cancer cells also consistently showed a significant positive association with TNM stage, T stage, N stage, and M stage (Figure S2C, Table S2). Therefore, metabolic^high^ *UBE2C+* cancer cells not only increased significantly with the RCC progression but also showed a dramatic increase in the tumor core (Figure S3C). This suggested that metabolic^high^ *UBE2C+* cancer cells may play a more important role in the initiation of RCC progression, which was further supported by existing research [12]. By studying the marker gene of cancer cells, we found that increased expression of *UBE2C* was significantly associated with poor survival outcome, which was consistent with the results of metabolic^high^ *UBE2C+* cancer cells. In addition, the marker gene *UBE2C* was significantly increased with TNM stage, T stage, N stage, and M stage (Figure S3D). In agreement with the results presented here, an investigation of publicly available protein data from “The Human Protein Atlas” indicated a worse prognosis for patients with high expression of *UBE2C* in renal cancer (Figure S4A). Immunohistochemical staining using an antibody targeting *UBE2C* (CAB011464) shows a differential expression pattern in renal cancer samples (Figure S4B). In proteomic data PDC000127, *UBE2C* protein abundance was significantly upregulated in tumor samples compared to normal tissues (Figure S4C). Functional analysis showed that metabolic^high^ *UBE2C+* cancer cells were significantly associated with G2M checkpoint, E2F targets, MYC targets, DNA repair, glycolysis, and cholesterol homeostasis gene sets. Metabolic^high^ *UBE2C+* cancer cells exhibited functional states associated with cell cycle, DNA repair, proliferation, and invasion (Figure S4D). For KEGG pathway, cell cycle, pyrimidine, glutathione, and nucleotide metabolism were significantly enriched in metabolic^high^ *UBE2C+* cancer cells. Differentially expressed genes in patients with high infiltration of metabolic^high^ *UBE2C+* cancer cells were significantly associated with pyrimidine metabolism, nucleotide metabolism, and ferroptosis (Figure 2E). In particular, the consistent results were observed in two independent RCC scRNA-seq validation cohorts (Figure S2D,E). Since tumor metabolism is controlled by changes in metabolite abundance, we collected metabolomic and transcriptomic data from RCC to reveal the relationship between metabolites and metabolic^high^ *UBE2C+* cancer cells. Multiple metabolites were significantly altered in RCC patients and showed a correlation with metabolic^high^ *UBE2C+* cancer cells and *UBE2C* (Figure 2F). For instance, cysteinylglycine disulfide, a downstream product of glutathione metabolism, was significantly more abundant in tumor samples and negatively correlated with metabolic^high^ *UBE2C+* cancer cells and *UBE2C*. This indicates that glutathione metabolism may play a potential role in metabolic^high^ *UBE2C+* cancer cells. Pyruvate and histamine were significantly enriched in tumor samples and showed a significant positive correlation with metabolic^high^ *UBE2C+* cancer cells and *UBE2C*. Studies have shown that cancer cells use glycolysis to rapidly produce energy from pyruvate, which in turn accelerates cancer growth [16]. These results suggested that metabolic reprogramming of metabolic^high^ *UBE2C+* cancer cells may promote multiple cancer processes, such as proliferation, migration, and invasion.

Additionally, we constructed cancer cell-specific gene regulatory networks using the pySCENIC algorithm to identify highly expressed TFs and their targets (Figure S4E) [17]. *BRCA1*, *E2F1*, *EZH2*, and *MXD3* had the highest expression and activity levels in the regulatory network of metabolic^high^ *UBE2C+* cancer cells. Functional analysis showed that top-ranked TF regulons of metabolic^high^ *UBE2C+* cancer cells were significantly enriched in cell cycle and DNA replication. Dysregulation of the cell cycle is one of the most important characteristics of tumor expansion and invasion, and *BRCA1*, *E2F1*, *EZH2*, and *MXD3* drove the cell cycle of cancer cells. To characterize the differentiation states among cancer cells, trajectory analysis was performed to decipher the cell trajectory. The result revealed that metabolic^high^ *UBE2C+* cancer cells represented a less differentiated or progression-associated tumor cell state (Figure 2G). Therefore, we considered metabolic^high^ *UBE2C+* cancer cells as a progression-associated malignant subpopulation in the tumor core region, which may play a more important role in the tumorigenesis, metabolism, and progression of RCC.

### 2.3. The Immune Landscape Revealing PLK1+ NK Cells, Plasma Cells, and CDC20+ Macrophages as RCC Progression-Associated Immune Cell Subpopulations

**Figure 3:**
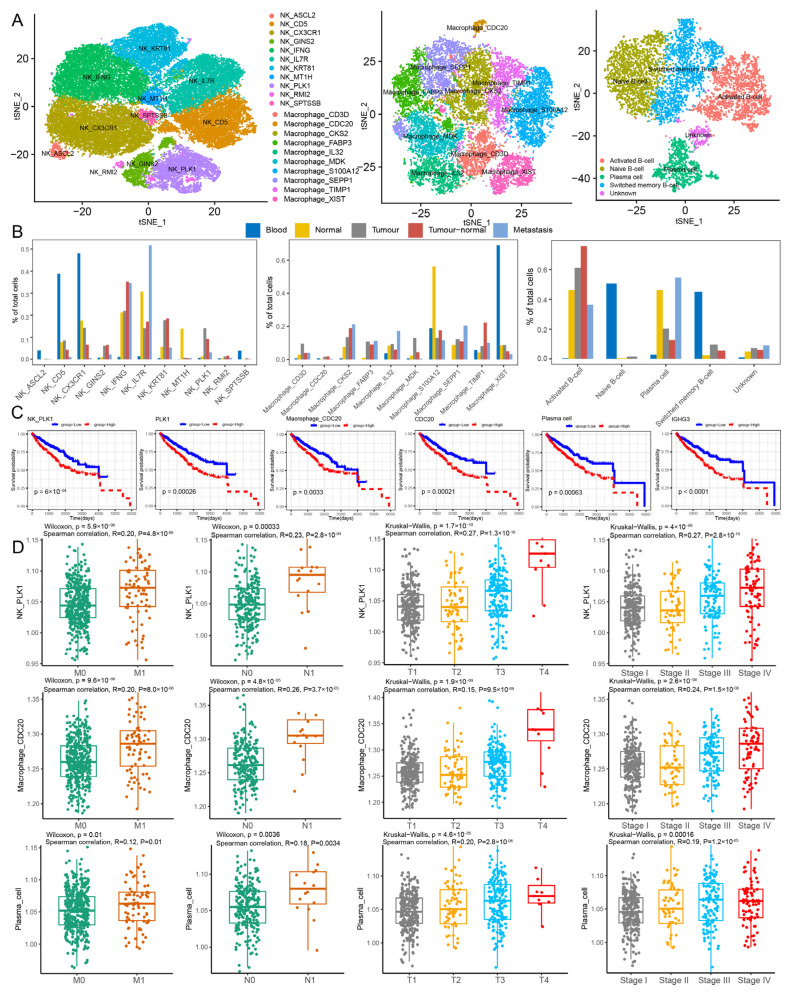
**The Immune Landscape of RCC.** (**A**) TSNE shows subpopulations of NK cells, B-cells, and Macrophage identified by marker gene expression. Dot colors refer to immune cell subpopulations. (**B**) Bar plot shows the proportion of cell subpopulations enriched in different tumor regions. (**C**) The Kaplan–Meier survival curves of RCC patients stratified by immune cell infiltration. Line colors refer to groups by cell abundance. (**D**) Comparison of absolute infiltration proportion of NK cells, B-cells and Macrophage in M classification, N classification, T classification, stage.

In order to more comprehensively understand the tumor immune microenvironment of cancer patients and reveal the function of immune cell subpopulations, we applied CPcellsubpopulation to identify cancer progression-associated immune cell subpopulations. T-cells (111,258) were further reclustered into seven subpopulations (Figure S5A,B). Regulatory T-cells (Tregs) and *CD8* T-cells were mainly distributed in the tumor core and tumor–normal interface, respectively (Figure S5C). *CD8* T-cells and Tregs were positively correlated with tumor size, tumor stage, and distant metastasis (*p* < 0.01; Figure S5D). Functional analysis showed that these cells were significantly correlated with interferon response, cell cycle, and inflammation characteristics (Figure S6B). NK cells (56,353) were further reclustered into 11 subpopulations (Figure 3A and Figure S6A). *PLK1+* NK and *IFNG*+ NK cells were mainly distributed in the tumor core and tumor–normal interface, respectively (Figure 3B). Patients with high infiltration of *PLK1+* NK cells and high *PLK1* expression showed a poor prognosis (Figure 3C). Furthermore, *PLK1+* NK cells and *PLK1* were positively correlated with distant metastasis, tumor size, tumor stage, and lymph node metastasis (Figure 3D and Figure S6B). The inhibitory receptors (*PDCD1*, *HAVCR2*, and *LAG3*) on *PLK1+* NK cells were highly expressed, and the activation receptors (*FCGR3A* and *NCR3*) on *PLK1+* NK cells were poorly expressed (Table S2), suggesting NK cell exhaustion and tumor immune evasion [18]. *PLK1+* NK cells were significantly correlated with oxidative phosphorylation, glycolysis, E2F targets, and G2M checkpoint, and exhibited functional states related to the cell cycle, proliferation, and metastasis (Figure S6C). B-cells (7365) were further reclustered into four subpopulations (Figure 3A and Figure S7A). Plasma cells were the main B-cell subpopulation in metastatic tissues (Figure 3B). Patients with high plasma cell infiltration and high expression of *IGHG3* showed poor survival outcomes (Figure 3C). Plasma cells and *IGHG3* showed a positive correlation with distant metastasis, tumor size, tumor stage, and lymph node metastasis (Figure 3D and Figure S7B). Functional analysis showed that plasma cells were significantly associated with epithelial-mesenchymal transition (EMT) and showed functional states of angiogenesis and hypoxia (Figure S7C). In addition, plasma cells overexpressed immune-modulatory genes (*PRDM1*, *PIM2*, and *CREB3L2*; Table S2), which may reflect possible immunoregulatory function [19,20,21]. Macrophages (18,407) were further reclustered into 10 cell subpopulations (Figure 3A and Figure S8A). Among them, *XIST*+ macrophages were almost all enriched in the blood. *CKS2*+ macrophages, *FABP3*+ macrophages, and *SEPP1*+ macrophages represented major macrophage subpopulations in the tumor core, tumor–normal interface and tumor metastasis (Figure 3B). Based on ssGSEA analysis, high infiltration of *CDC20*+ macrophages was associated with poor prognosis in patients (Figure 3C). Further analysis showed that *CDC20*+ macrophages were positively correlated with tumor size, tumor stage, lymph node metastasis, and distant metastasis (Figure 3D and Figure S8B). Functional analysis showed that *CDC20*+ macrophages were significantly correlated with mitotic spindle, G2M checkpoint, E2F targets, glycolysis, and MYC targets, and showed functional states of cell cycle, proliferation, and DNA damage (Figure S8C). *CDC20*+ macrophages were characterized by *CCL4* and *FN1* expression (Table S2), which may reflect an M2 phenotype and may be related to cancer metastasis [22]. Similarly, among five monocyte subpopulations (22,053) and four dendritic cell subpopulations (4948), the infiltration of *CD3E*+ monocytes was found to be increased gradually from stage I to stage IV and T1 to T4 (Figure S9). The infiltration level of dendritic cells was significantly higher in patients with stage III and IV disease (Figure S10). Functional analysis showed that *CD3E*+ monocytes and dendritic cells displayed functional states of stemness, angiogenesis, and proliferation. These results suggest that *PLK1+* NK cells, plasma cells, and *CDC20*+ macrophages as RCC progression-associated immune cell subpopulations, may play important roles in the remodeling of TME, which potentially promotes the formation and progression of RCC.

### 2.4. Cell–Cell Interaction Between Cancer Progression-Associated Cancer Cells and Immune Cells Revealed by Spatial Transcriptomics

**Figure 4:**
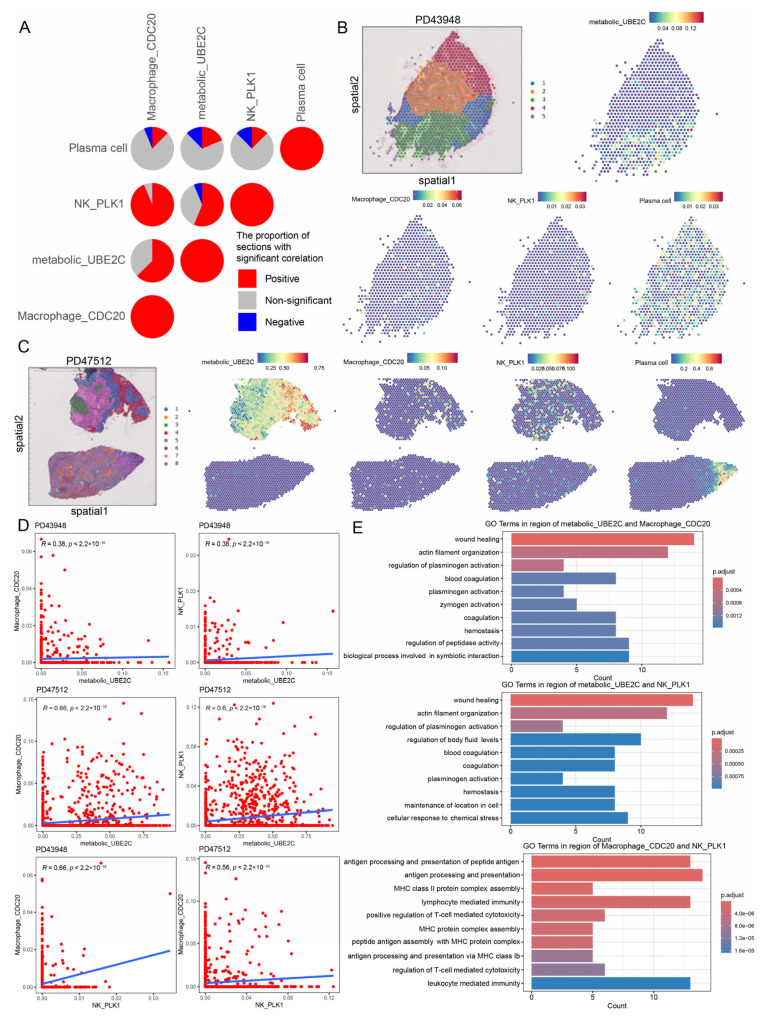
**Co-localization of tumor cells and immune cells revealed by spatial transcriptomics.** (**A**) The proportion of spatial transcriptomics sections with positive (Spearman correlation; R > 0.2 and *p* < 0.05, in red), negative (R < −0.2 and *p* < 0.05, in blue), or non-significant (gray) correlation for the infiltration of pairwise cell subpopulations in 16 spatial transcriptomics sections. (**B**) Clustering of ST points and spatial feature plots of signature score of metabolic^high^ *UBE2C+* cancer cells and RCC progression-associated immune cell subpopulations in the tumor core tissue section (PD43948). (**C**) Clustering of ST points and spatial feature plots of signature score of metabolic^high^ *UBE2C+* cancer cells and risk TME immune cell subpopulations in the tumor–normal interface tissue section (PD47512). (**D**) The Spearman correlation of cancer cells and immune cells. (**E**) Gene ontology (GO) terms of genes significantly enriched in the co-localization regions of cell subpopulation.

The spatial distribution of TME cells and their density changes are important in the malignant progression of cancers. To map the spatial architecture of RCC, we performed ST analysis of 11 tumor–normal interface tissue sections and five tumor core tissue sections from eight RCC patients. Based on the STAGATE algorithm, we performed cluster analysis (Figure S11) [23]. For example, we classified the spots into nine clusters for both the tumor core sample (PD47171) and the tumor–normal interface sample (PD43824). The RCTD method was applied to categorize the mixed cell types of each spot. Metabolic^high^ *UBE2C+* cancer cells and RCC progression-associated immune cell subpopulations were co-located in the same spots of the tumor core and tumor–normal interface sections (Figures S12–S14). High correlations were found between metabolic^high^ *UBE2C+* cancer cells and *PLK1+* NK cells across nine tissue sections, as well as between metabolic^high^ *UBE2C+* cancer cells and *CDC20*+ macrophages across 10 tissue sections (Figure 4A). For example, in the tumor core tissue section (PD43948), metabolic^high^ *UBE2C+* cancer cells were co-located with *PLK1+* NK cells, plasma cells, and *CDC20*+ macrophages in regions one and three (Figure 4B). Metabolic^high^ *UBE2C+* cancer cells, *PLK1+* NK cells, and *CDC20*+ macrophages showed a significantly positive correlation. In the tumor–normal interface tissue section (PD47512), metabolic^high^ *UBE2C+* cancer cells were co-located with *PLK1+* NK cells and *CDC20*+ macrophages in region 4 (Figure 4C). Moreover, metabolic^high^ *UBE2C+* cancer cells were significantly positively correlated with *PLK1+* NK cells and *CDC20*+ macrophages (Figure 4D). The co-localization of cancer progression-associated cancer cells and immune cells indicated that there may be a spatial association between them. Subsequently, we further identified differentially expressed genes within the cell co-localization regions of each slice and identified recurrent genes, focusing on the functions enriched by the top 1% of genes. Further functional analysis revealed wound healing, the regulation of plasminogen activation and actin filament organization were significantly enriched in the co-localization regions of metabolic^high^ *UBE2C+* cancer cells and *CDC20*+ macrophages (Figure 4E). Similar functions were found in regions where *PLK1+* NK cells and metabolic^high^ *UBE2C+* cancer cells were co-located. Studies have shown that tumors use the wound-healing response to enhance their survival and growth [24]. Immune-, metabolism-, and proliferation-related gene sets were significantly positively correlated with metabolic^high^ *UBE2C+* cancer cells, *PLK1+* NK cells, and *CDC20*+ macrophages in the tissue sections (Figure S15A). Gene sets such as G2M checkpoints, MYC targets, E2F targets, glycolysis, and inflammatory response showed higher activity in the regions where metabolic^high^ *UBE2C+* cancer cells, *PLK1+* NK cells, and *CDC20*+ macrophages were co-located in the tissue sections (Figure S15B,C). Furthermore, *PLK1+* NK cells and *CDC20*+ macrophages showed a significant positive correlation in 15 tissue sections (Figure 4A). For instance, *PLK1+* NK cells and *CDC20*+ macrophage showed a significant positive correlation in PD43948 and PD47512 (Figure 4D). Functional analysis revealed that immune-related functions, such as antigen processing and presentation, and lymphocyte-mediated immunity, were significantly correlated with the co-localization regions of *PLK1+* NK cells and *CDC20*+ macrophages (Figure 4E and Figure S15B,C). Previous studies have shown that macrophages interact with NK cells to optimize the anti-tumor function [25]. These results suggest a spatial association between metabolic^high^ *UBE2C+* cancer cells and RCC progression-associated immune cell subpopulations, consistent with potential functional interactions within the tumor microenvironment.

### 2.5. Multiregional Tumor–Immune Communication Networks

**Figure 5:**
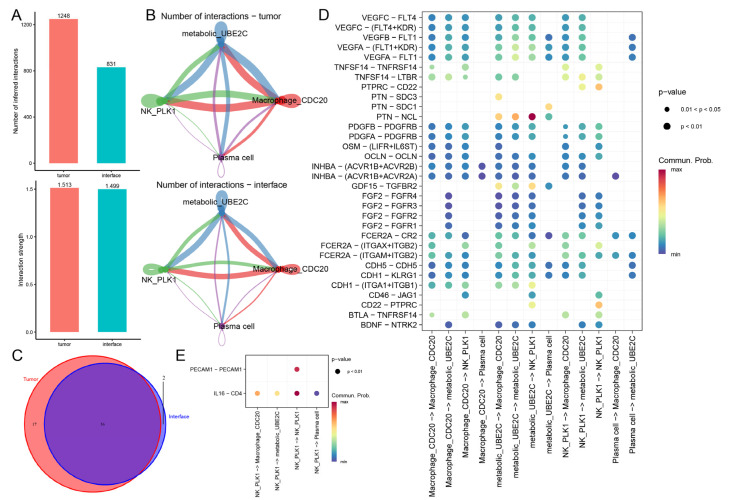
**Specific communication patterns between cancer cells and immune cells in the tumor core and tumor–normal interface.** (**A**,**B**) The landscape of cell communications between cancer cells and progression-associated immune cells in the tumor core and the tumor–normal interface, respectively. (**C**) Venn plot displayed the numbers of specific interactions in the tumor core and the tumor–normal interface. (**D**,**E**) Bubble plots displayed the communication probability of specific signaling pathways between cancer cells and progression-associated immune cells in the tumor core (**D**) and the tumor–normal interface (**E**).

Cell signaling and communication between cancer cells and immune cells are crucial for the cancer progression and metastasis. Using CellChat algorithm, we evaluated the cell–cell communications within the TME. Although the number of interactions between cell subpopulations was higher in the tumor core, the strength of interactions among different regions was comparable (Figure 5A,B). Then we identified the specific ligand–receptor pairs that were present in the tumor core and tumor–normal interface tissue (Figure S16A). There were more specific signaling pathways in the tumor core than in the tumor–normal interface (Figure 5C). Among them, 74.7% of the signaling pathways coexisted in the tumor core tissue and the tumor–normal interface. A total of 22.7% of the specific signaling pathways (*ACTIVIN*, *BTLA*, *CD23*, *CDH1*, *CDH5*, *LIGHT*, *OCLN*, *OSM*, *PDGF*, *VEGF*, *CD46*, *FGF*, *NT*, *CD22*, *CD45*, *GDF*, *PTN*) were present in the tumor core tissue, while 3.6% of the signaling pathways (*IL16*, *PECAM1*) were specific to the tumor–normal interface. The results suggested regional heterogeneity in the ligand–receptor pairs. The *VEGF* pathway was predicted to be activated between metabolic^high^ *UBE2C+* cancer cells and immune cells in the tumor core, suggesting that it may be involved in RCC progression (Figure 5D). Remarkably, in the tumor core tissue, ligands (*VEGFA*, *VEGFB*, and *VEGFC*) were highly expressed in metabolic^high^ *UBE2C+* cancer cells, while receptors (*FLT1* and *KDR*) were overexpressed in *PLK1+* NK cells (Figure S16B). The ligand–receptor pairs (*VEGFA-FLT1*, *VEGFA-FLT1/KDR*, *VEGFB-FLT1*, *VEGFC-FLT4*, *VEGFC-FLT4/KDR*) showed a significant positive correlation in the ST data (Figure S16D). The *GDF* and *PTN* signaling pathways (*GDF15-TGFBR2, PTN-NCL*) were predicted to be enriched between metabolic^high^ *UBE2C+* cancer cells and progression-associated immune cells in the tumor core. Ligands (*GDF15* and *PTN*) were highly expressed in metabolic^high^ *UBE2C+* cancer cells, and receptors (*TGFBR2* and *NCL*) were overexpressed in *PLK1+* NK cells and *CDC20*+ macrophages (Figure S16B). The ligands and receptors of *GDF15-TGFBR2* and *PTN-NCL* were significantly positively correlated in eleven and eight sections, respectively (Figure S16D). Studies have shown that *PTN-NCL* plays an important role in the growth and angiogenesis of prostate cancer [26]. *GDF15-TGFBR2* is related to immunosuppression in the gastric cancer [27]. Of note, metabolic^high^ *UBE2C+* cancer cells may interact with *CDC20*+ macrophages through the *PTN-SDC3* pair, and the expression levels of *PTN* and *SDC3* were higher in metabolic^high^ *UBE2C+* cancer cells and *CDC20*+ macrophages, respectively (Figure S16B). There was a significant positive correlation between *PTN* and *SDC3* in eight sections (Figure S16D). *PTN* maintains the M2-like phenotype of macrophages through *SDC3* [28]. The ligand–receptor pairs (*CD22-PTPRC, PTPRC-CD22*) were predicted between metabolic^high^ *UBE2C+* cancer cells and *PLK1+* NK cells in the tumor core, suggesting that these interactions may be involved in the immune response against tumors [29]. In the tumor–normal interface tissue, the *IL16* pathway (*IL16-CD4*) was inferred to be enriched between *PLK1+* NK cells and other cell subsets (Figure 5E). *IL16* was mainly expressed in *PLK1+* NK cells, and *CD4* was overexpressed in *CDC20*+ macrophages (Figure S16C). *IL16* and *CD4* showed a significant positive correlation in the 13 ST sections (Figure S16D). Previous studies have shown that overexpression of secreted *IL-16* recruits *CD4*+ tumor-promoting macrophages in breast cancer [30]. Collectively, these results suggest that the predicted interaction network between metabolic^high^ *UBE2C+* cancer cells and RCC progression-associated immune cell subpopulations in different tissues, may provide insights into the remodeling of immunosuppressive microenvironments and cancer progression in RCC.

### 2.6. Identification of Critical Regulators, Candidate Drugs, and the Efficacy of Immunotherapy in Metabolic^high^ UBE2C+ Cancer Cells

**Figure 6:**
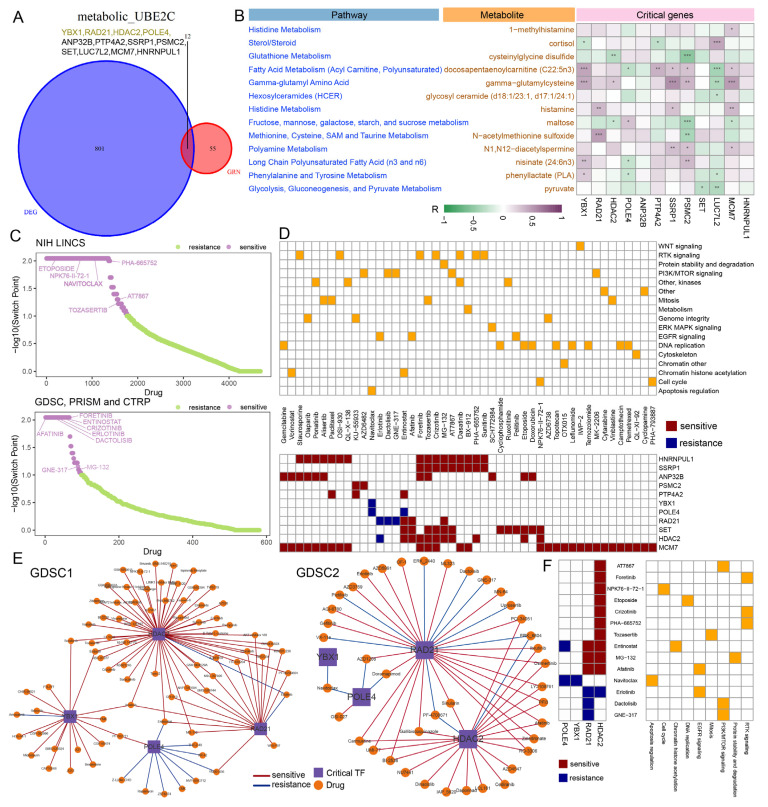
**Potential drugs targeting critical TFs.** (**A**) The Venn diagram shows the intersection of critical genes and differentially expressed genes, yellow represents TFs, black represents target genes. (**B**) Correlation between critical genes and metabolites. (**C**) Scatter plots of switch point of a certain drugs in the metabolic^high^ *UBE2C+* cancer cells. (**D**) A total of 47 potential drugs identified by both strategies and their associated signaling pathways. Square colors indicate predicted sensitivity or resistance to the indicated drugs. (**E**) The network of TFs and TF-related drugs. Line colors indicate predicted sensitivity or resistance to the indicated drugs. Dot shapes refer to TF or drug. (**F**) A total of 14 potential drugs identified in both strategies and their associated signaling pathways. ***: *p* < 0.001; **: *p* < 0.01; *: *p* < 0.05.

To further elucidate the regulatory mechanisms of tumor cells, a cell-specific gene regulatory network (GRN) was constructed using SCENIC in metabolic^high^ *UBE2C+* cancer cells. The critical genes were identified in the GRN using centrality indices of the network [31]. A total of 67 critical genes were identified in the metabolic^high^ *UBE2C+* cancer cells (Figure S17A). Next, we identified 12 critical genes that were specifically differentially expressed in the metabolic^high^ *UBE2C+* cancer cells, including four TFs and eight target genes (Figure 6A). Through metabolic analysis, RAD21 was significantly positively correlated with histamine and N-acetylmethionine sulfoxide (Figure 6B). Histamine is associated with tumor progression-related metabolic pathways in RCC [32]. Furthermore, consistent with the metabolic^high^ *UBE2C+* cancer cells, docosapentaenoylcarnitine, as a metabolite associated with fatty acid metabolism, showed a significant positive correlation with *YBX1, PSMC2, PTP4A2, SSRP1,* and *MCM7*. Transcriptional activation of *YBX1* promotes colorectal cancer growth by fatty acid metabolism [33]. *YBX1, HDAC2, PSMC2, SSRP1,* and *MCM7* were significantly positively correlated with gamma-glutamylcysteine. Gamma-glutamylcysteine is strongly implicated in tumor growth and migration [34]. These results suggested that the critical genes may play a key role in the metabolic reprogramming of metabolic^high^ *UBE2C+* cancer cells.

To determine whether critical genes can contribute to clinical therapy, we predicted the potential drug responses of RCC based on IC50 values. A total of 156 and 120 significantly associated compounds were identified from the GDSC1 and GDSC2 databases, respectively (Figure S17B). Meanwhile, the Beyondcell algorithm, as another strategy, was used to predict candidate drugs for metabolic^high^ *UBE2C+* cancer cells. We identified 1762 compounds from the Library of Integrated Network-based Cellular Signatures (LINCS), and 124 compounds from the Cancer Cell Line Encyclopedia (CCLE), the Genomics of Drug Sensitivity in Cancer (GDSC), and the Cancer Therapeutic Response Portal (CTRP) (Figure 6C). Finally, a total of 47 compounds were identified by both strategies (Figure 6D). For instance, entinostat, targeting chromatin histone acetylation, was significantly associated with *PTP4A2*, *SET*, *RAD21*, *POLE4*, and *HDAC2*. Tozasertib, targeting mitosis, was significantly associated with *HNRNPUL1*, *SSRP1*, *ANP32B*, *SET*, *MCM7*, and *HDAC2*. Studies have shown that entinostat and tozasertib can activate anti-tumor immunity to inhibit tumor growth [35,36]. These compounds represent candidate therapeutic drugs for metabolic^high^ *UBE2C+* cancer cells. Since transcription factors play a crucial role in the occurrence, progression, and treatment of cancer, we further investigated four critical TFs to evaluate prognostic relevance and their potential utility in clinical treatment. Critical TFs and other clinicopathological factors, including age, T stage, N stage, M stage and overall TNM stages, were used as covariates. The results revealed *RAD21* as a protective factor and an independent prognostic factor for RCC (Figure S17C). Studies have shown that *RAD21* was downregulated in kidney cancer samples and indicated poor prognosis [37]. Next, by integrating the two strategies, we identified 14 compounds that were significantly correlated with the critical TFs (Figure 6E,F). For example, entinostat, MG-132, and afatinib were significantly negatively associated with *RAD21*. Erlotinib, dactolisib, and GNE-317 were significantly positively associated with *RAD21*. Studies have shown that these drugs have a favorable therapeutic effect in cancer treatment [38,39,40]. These results suggested that *RAD21* and these compounds may be candidate therapeutic targets and drugs for future RCC targeted therapy.

To further evaluate the clinical relevance of metabolic^high^ *UBE2C+* cancer cells, we performed additional analyses focusing on disease recurrence and therapeutic response. First, using the TCGA-KIRC cohort, we incorporated disease-free survival (DFS) and recurrence-related clinical information. Patients with higher abundance of metabolic^high^ *UBE2C+* cancer cells exhibited significantly shorter DFS (Figure S17D). Consistently, metabolic^high^ *UBE2C+* cancer cells were significantly enriched in patients who experienced disease recurrence, suggesting their association with disease progression (Figure S17E). Next, we analyzed two independent RCC cohorts of treated patients, including RCC treatment cohort (JAVELIN-Renal-101) and the immunotherapy cohort (RCC-Braun2020). In both cohorts, a higher abundance of metabolic^high^ *UBE2C+* cancer cells was significantly associated with worse clinical outcomes (Figure S17F). Importantly, patients who did not respond to immunotherapy exhibited significantly higher levels of metabolic^high^ *UBE2C+* cancer cells compared to responders (Figure S17G). These findings suggest that metabolic^high^ *UBE2C+* cancer cells may be associated with reduced therapeutic response, representing potential therapeutic targets in RCC treatment.

## 3. Discussion

Tumor heterogeneity represents an ongoing challenge in the field of cancer treatment and may be a key factor in treatment success [41]. In this study, CPcellsubpopulation, a comprehensive computational framework, was developed to identify cancer progression-associated cell subpopulations. Using this framework, we identified 46 tumor cell subpopulations linked to disease progression across nine distinct cancer types. In renal cell carcinoma, we consistently identified a *UBE2C+* cancer cell subpopulation that is strongly associated with clinical progression and patient prognosis across multiple datasets.

Cancer cell status has been used to study transcriptional heterogeneity among malignant cells in tumors [13]. Here, we performed scRNA-seq analysis across 22 RCC patients and identified a catalog of gene modules whose expression defines recurrent cancer cell states including metal response, ATP synthesis, metabolic, immune response, differentiation, ECM, stress response, and protein response modules. ATP synthesis and metabolic modules were defined and shared by multiple RCC cell subpopulations in our study. Metabolic^high^ *UBE2C+* cancer cells, as RCC progression-associated cell subpopulations, have been identified in multiple RCC single-cell cohorts, and increased significantly with the progression of tumor TNM stage. Patients with high abundance of metabolic^high^ *UBE2C+* cancer cells and high expression of *UBE2C* showed the worse prognosis. Genome-wide CRISPR-Cas9 screening data from the Cancer Dependency Map (DepMap) database were utilized to evaluate the functional impact of *UBE2C* on cell viability [42]. Gene effect scores indicated that the knockdown of *UBE2C* influenced cell viability in all RCC cell lines (Figure S18). In addition, metabolic^high^ *UBE2C+* cancer cells exhibited a less differentiated or progression-associated state, which may represent an early progression-associated malignant state. These results suggest that metabolic^high^ *UBE2C+* cancer cells, as oncogenic subpopulation, may be potential cancer progression promoters. Next, we identified metabolic^high^ *UBE2C+* cancer cells and three cancer progression-associated immune cell subpopulations by utilizing CPcellsubpopulation. By integrating ST data, RCC progression-associated cell subpopulations exhibit spatial co-localization patterns, which may indicate spatial association between cancer cells and immune cells. Cell communication further identified specific ligand–receptor pairs between cancer cells and immune cells in different tissues. *GDF*, *PTN*, and *IL16* signaling pathways were enriched in the tumor core and tumor–normal interface, respectively. Therefore, an in-depth understanding of the interaction and communication between RCC progression-associated cancer cells and immune cells in different tissues will help to develop targeted therapies for RCC.

Based on metabolic^high^ *UBE2C+* cancer cells, we constructed cell-specific GRN and identified 12 critical genes that were significantly correlated with energy, lipid, and amino acid metabolism. Critical genes may play an important regulatory role in tumor cell proliferation and metabolic reprogramming. By genome-wide CRISPR-Cas9 screening data, the knockdown of critical TFs (*RAD21*, *POLE4* and *YBX1*) and genes (*SSRP1*, *PSMC2*, *MCM7*, *LUC7L2* and *PTP4A2*) markedly influenced cell viability in more than 80% of RCC cell lines (Figure S18), indicating the potential roles of critical genes in tumor cell proliferation. Next, we explored four critical TFs and their associated fourteen compounds through two strategies. Among them, *RAD21*, as an independent prognostic factor, was significantly associated with these compounds, such as entinostat, erlotinib, and dactolisib. Entinostat has been used to treat breast cancer and non-small cell lung cancer [43,44]. These findings highlight the potential of our framework to identify candidate therapeutic targets and compounds.

By integrating several well-established analytical approaches, including scRNA-seq clustering, ssGSEA, survival analysis, and correlation analysis, CPcellsubpopulation systematically identified cancer progression-associated cell subpopulations. These subpopulations serve as potential biomarkers associated with cancer prognosis and progression. Despite these findings, several limitations should be acknowledged. Although our analyses provide a comprehensive strategy to identify potential biomarkers and candidate therapeutic compounds for RCC, functional validation is still required to truly propose novel drugs for RCC. Furthermore, all analyses were conducted based on publicly available datasets, including scRNA-seq, bulk RNA-seq, and spatial transcriptomics data. While these datasets are of high quality and widely used, the incorporation of additional RCC cohorts and experimental validation in future studies will further strengthen the novelty and robustness of our findings.

## 4. Materials and Methods

### 4.1. Data Collection and scRNA-seq Data Preprocessing

To develop cell subpopulation-specific genes, we screened scRNA-seq datasets that contained at least 15,000 cells after quality control. Ten pan-cancer scRNA-seq datasets, containing 1,016,892 cells and 173,842 cancer cells, were collected from breast cancer, colorectal cancer, muscle invasive bladder cancer, liver hepatocellular carcinoma, lung adenocarcinoma, pancreatic adenocarcinoma, skin cutaneous melanoma, and renal cell carcinoma (Table S3). We collected data on gene expression, survival time, and clinicopathological features (including M, N, T, and overall TNM stage classifications) for the corresponding nine cancers from The Cancer Genome Atlas (TCGA, https://portal.gdc.cancer.gov, accessed on 20 January 2025, Table S4). For RCC datasets, we obtained three scRNA-seq datasets, comprising 421,952 cells from 22 RCC patients. The Li et al. dataset was selected as the initial model due to its multi-regional sampling design and availability of matched ST data [45]. The Bi et al. and Krishna et al. datasets, as independent validation cohorts, were used to validate the reproducibility of identified cell subpopulations [46,47]. We compared demographic information and clinical characteristic across the three datasets using the Kruskal–Wallis test and Fisher’s exact test. The validation cohorts showed similar characteristics of the initial model, including age, sex, and tumor stage (*p* > 0.05). Further quality control, feature selection, dimension reduction, unsupervised clustering, and differential expression analyses were performed using the Seurat R package (v 5.4.0) [48]. ST datasets for RCC were obtained, including 11 tumor–normal interface tissue sections and five tumor core tissue sections from eight RCC patients [45]. Moreover, RNA-seq data and clinical data of 607 RCC samples were obtained from TCGA. We obtained transcriptome and clinical data on RCC patients receiving PD-1 blockade immunotherapy from Braun et al., including 311 RCC samples (RCC-Braun2020) [49]. JAVELIN-Renal-101, as RCC first-line treatment cohort, was acquired from Motzer et al., including 726 samples [50]. Metabolomic and matched transcriptomic data of RCC were collected, including 67 tumor samples and 47 normal samples [51]. The processed proteomic data of RCC (PDC000127) was collected from the Clinical Proteomic Tumor Analysis Consortium (CPTAC, https://pdc.cancer.gov/pdc/, accessed on 29 April 2026), including 81 normal and 103 tumor cases [52,53]. We obtained 14 different functional states of cancer cells from the CancerSEA database [54].

### 4.2. Identification of Cell Types

We manually recorded canonical marker genes for major cell types from ACT (http://biocc.hrbmu.edu.cn/ACT/, accessed on 5 March 2025) [55]. Based on the markers outlined above, we used ScType (https://github.com/IanevskiAleksandr/sc-type, accessed on 7 March 2025), a fully automated and ultrafast cell type identification method, to assign cell types to each cluster [56]. We applied the Cancer-Finder (v 1.0.1) to extract and transform features, and to infer malignant cells. A result value, ranging from 0 to 1, is calculated to differentiate between malignant and non-malignant cells, with a default threshold of 0.5 [11].

### 4.3. Identification of Cancer Progression-Associated Cell Subpopulations by CPcellsubpopulation

To identify cancer progression-associated cell subpopulations, CPcellsubpopulation was designed. The detailed processes are as follows. Firstly, within the scRNA-seq data, cells are classified into major cell types and subsequently subjected to further sub-clustering. Based on specific cellular signature markers, distinct cell subpopulations are thereby identified. For each cell subpopulation, we identify those genes whose expression levels are statistically higher compared to other cells (adjusted *p*-value < 0.05 and log2FC > 1). These genes are then defined as the cell subpopulation-specific gene set. Second, with cell subpopulation-specific gene sets extracted from the scRNA-seq data, single-sample gene set enrichment analysis (ssGSEA) is employed. This analysis is used to predict the abundance of each cell subpopulation, recorded as ssGSEA scores, within large-scale datasets sourced from TCGA. Thirdly, to evaluate the clinical relevance of these cancer cell subpopulations, samples are stratified into high-infiltration and low-infiltration groups according to their ssGSEA scores. Subsequently, the impact of different cell infiltration levels on patient prognosis is investigated. A *p*-value less than 0.05 is considered evidence that the cell subpopulation is associated with prognosis. Fourthly, to explore whether cell subpopulations are involved in cancer progression, the Wilcoxon test and Kruskal–Wallis test are used to compare the differences of ssGSEA scores across clinicopathological features (including M, N, T, and overall TNM stage classifications). *p* < 0.05 indicates that the cell subpopulation displays a significant difference in M, N, T, and overall TNM stages. Then, Spearman’s correlation analysis is performed to assess the relationship between these clinicopathological features and cell subpopulation infiltration. |R| > 0 and *p* < 0.05 were considered a significant correlation. Finally, we define cancer progression-associated cell subpopulations as those whose infiltration levels are correlated with patient prognosis as well as the progression of TNM, T, N, and M stages. The CPcellsubpopulation facilitates the identification of cell subpopulations involved in cancer formation and development.

### 4.4. Expression Programs of Intratumoral Heterogeneity in RCC

To explore underlying intratumoral expression signatures of cell populations in RCC, non-negative matrix factorization (NMF, v 0.28) was performed separately on the identified malignant cells of each sample. Briefly, for malignant cells of each sample, we first normalized the expression counts using the Seurat NormalizeData() function with default parameter settings. We selected highly variable genes (HVGs) using the Seurat FindVariableFeatures() function and only focused on the 2000 HVGs in downstream analysis. As the application of NMF requires a parameter K that influences the results, we ran NMF using different values (K = 2–10), thereby generating 91 programs for 22 patients. Each NMF program was summarized by the top 50 genes based on NMF coefficients. We next clustered the robust NMF programs according to Jaccard similarity. The program with the maximal number of considerable overlaps was selected as a potential founder of a new cluster. The module for the cluster was defined by the genes that repeatedly appeared between programs. We ranked each gene according to the number of programs observed, and the top 10 genes were selected. Significantly enriched Gene Ontology and gene sets from MsigDB were used to characterize the module function.

### 4.5. Spatial Transcriptomics Data Analysis

ST slides included 11 tumor–normal interface tissue sections and five tumor core tissue sections from eight RCC patients. Based on the STAGATE algorithm (v 1.0.1), we integrated spatial information and gene expression profiles to learn low-dimensional latent embeddings to accurately identify spatial domains [23]. Specifically, STAGATE first constructs the spatial neighbor network (SNN) based on the relative spatial locations of spots, and the cell type-aware SNN based on the pre-clustering of gene expression, respectively. The spatial similarity is calculated by integrating SNN and cell type-aware SNN with equal weights (the default weight is set to 0.5). Next, STAGATE employs the graph attention autoencoder to learn low-dimensional latent embeddings that jointly capture spatial context and gene expression patterns. Through an attention mechanism, the algorithm adaptively learns the similarity between neighboring spots. Finally, the learned latent embeddings are used as input for clustering with the mclust algorithm to identify spatial domains. Robust cell type decomposition (RCTD) was used to map the cell types found in the reference scRNA-seq dataset to spatial transcriptomic data [57]. Following the standard RCTD analysis pipeline on the spatial transcriptomics data, we obtained the probabilities of cell types for each spot in the ST slice.

### 4.6. Transcription Factor Regulon Analysis

SCENIC was used to infer gene regulatory networks. We therefore log2-transformed the normalized counts of each cell lineage and used them with pySCENIC to perform co-expression analysis and TF-binding motif enrichment analysis between TFs and target genes [17]. Cytoscape 3.9.1 was used to visualize the network.

### 4.7. Pseudo-Time Trajectory Inference

The R package Monocle2 (v 2.36.0) was used to infer cell differentiation trajectories on scRNA-seq data [58]. Graph-learning-based dimensionality reduction analysis was performed by using the Monocle2 package to visualize the distribution of cancer cell subpopulations with cell differentiation.

### 4.8. Identification of Critical Genes in Gene Regulatory Network

Based on the GRN from the cancer cell subpopulation, we used the R package igraph (v 2.2.1) to separately compute the centrality indices of the network to measure the importance of the network nodes, including degree, betweenness, eigenvalue, PageRank, and closeness [31,59]. We used a published bioinformatics method to generate different priorities for the five centrality indexes of the network. Order statistics were applied to fuse these centrality indices to prioritize the node genes in the network, and Q statistic was calculated for each gene. Top 1% genes ranked by the Q statistic were considered critical genes. The Q statistic was calculated using the following formula:

(1) $$V_{k}=\sum_{j=1}^{k}{\left(-1\right)}^{j-1}\frac{V_{k-1}}{j!}r_{N-k+1}^{j}$$

(2) $$Q(r_{1},r_{2},\ldots ,r_{i},\ldots ,r_{N})=N!V_{N}$$

where $r_{i}$ is the rank ratio for centrality index i, *N* is the number of centrality index used, $r_{0}$ = 0, and $V_{0}\;=\;1$.

### 4.9. Functional Enrichment Analysis

The R package clusterProfiler (4.16.0) was used to perform GO and KEGG enrichment analysis. Hallmark gene sets were downloaded from the MSigDB of Broad Institute (https://www.gsea-msigdb.org/gsea/index.jsp, accessed on 15 March 2025), containing 50 gene sets. To assign pathway activity estimates to each cell, we performed GSVA to the single-cell expression matrix using standard settings in the GSVA package.

### 4.10. Cell–Cell Communication Analysis

We assessed differences in putative cell–cell communication modules between cell types for samples at each tissue by integrating the gene expression data through CellChat (v 2.2.0) [60]. Following the standard CellChat pipeline, we used the default CellChatDB as the ligand–receptor database and then inferred cell type-specific communication by identifying overexpressed ligands or receptors in one cell group and then identifying enhanced ligand–receptor interactions when either the ligand or receptor was overexpressed.

### 4.11. Sensitivity Analysis of Anticancer Drugs

We mainly used a ridge regression-based method of the R package oncoPredict (v 1.2) to predict the IC50 values of potential drug responses [61]. The transcriptomic data of drug responses from the GDSC1 and GDSC2 databases were utilized as training sets. We used the calcPhenotype function to calculate the IC50 values for each corresponding drug and calculated Spearman’s correlation between the expression of 12 critical genes and the IC50 values of compounds (|cor| > 0.3 and FDR < 0.05). Then, the R package Beyondcell (v 2.2.2) was used to identify drug sensitivity from scRNA-seq data [62]. The drug sensitivity signatures collection (SSc) and perturbation susceptibility (PSc) collection from Beyondcell was used. The beyondcell score for each cell–drug pair and the switch point (SP) for each drug were calculated by the bcScore function with default parameters. In brief, SP represents that the cells are sensitive (SP = 0) or resistant (SP = 1) to the drug.

### 4.12. Statistical Analyses

All statistical analyses were implemented in R version 4.5.1. The statistical significance for variables between two groups was estimated by Wilcoxon rank sum test. In addition, for variables in more than two groups, the Kruskal–Wallis test was used. The significance of differences between survival curves was determined using the log-rank test.

## Data Availability

The single-cell RNA sequencing data for pan-cancer were downloaded from Gene Expression Omnibus (GEO, https://www.ncbi.nlm.nih.gov/geo/, accessed on 24 January 2025), Single Cell PORTAL (https://singlecell.broadinstitute.org/single_cell, accessed on 26 January 2025), SRA (https://www.ncbi.nlm.nih.gov/sra/, accessed on 30 January 2025), and the Mendeley Data (https://data.mendeley.com, accessed on 2 February 2025). The spatial transcriptomics data for RCC that support the findings of this study are available from the Mendeley Data. The bluk RNA-seq data for pan-cancer was obtained from The Cancer Genome Atlas database (TCGA, https://portal.gdc.cancer.gov, accessed on 20 January 2025). PD-1 blockade immunotherapy transcriptome data was obtained from Braun et al. RCC treatment cohort was obtained from Motzer et al. Proteomic data was collected from the CPTAC (https://pdc.cancer.gov/pdc/, accessed on 29 April 2026). The R code was used in the analysis of the data. All code generated for analysis is available from the authors upon request.

## Supplementary Materials

The following supporting information can be downloaded at: https://www.mdpi.com/article/10.3390/ijms27104456/s1.

## Abbreviations

RCC	renal cell carcinoma
ST	spatial transcriptomics
TME	tumor microenvironment
scRNA-seq	single-cell RNA sequencing
ssGSEA	single-sample gene set enrichment analysis
BRCA	breast cancer
LUAD	lung adenocarcinoma
SKCM	skin cutaneous melanoma
NMF	non-negative matrix factorization
GRN	gene regulatory network
LINCS	library of integrated network-based cellular signatures
CCLE	cancer cell line encyclopedia
CTRP	cancer therapeutic response portal
GDSC	genomics of drugs sensitivity in cancer
OS	overall survival
TF	transcription factor
TCGA	the cancer genome atlas
HVG	highly variable gene
RCTD	robust cell type decomposition
SSc	sensitivity signatures collection
PSc	perturbation susceptibility collection
ECM	extracellular matrix
EMT	epithelial-mesenchymal transition
DFS	disease-free survival
DepMap	cancer dependency map
CPTAC	clinical proteomic tumor analysis consortium
SNN	spatial neighbor network
