# Supplementary material for: A Multi-Omics Framework Reveals Tumor Heterogeneity and Predicts Therapeutic Targets in Renal Cell Carcinoma
Source: Int J Mol Sci. 2026 May 15;27(10):4456. doi: 10.3390/ijms27104456 (PMC13207066; doi:10.3390/ijms27104456)
Supplement: Supplementary file 1 [file ijms-27-04456-s001.zip › ijms-4280974-supplementary.pdf]

SUPPLEMENTARY INFORMATION

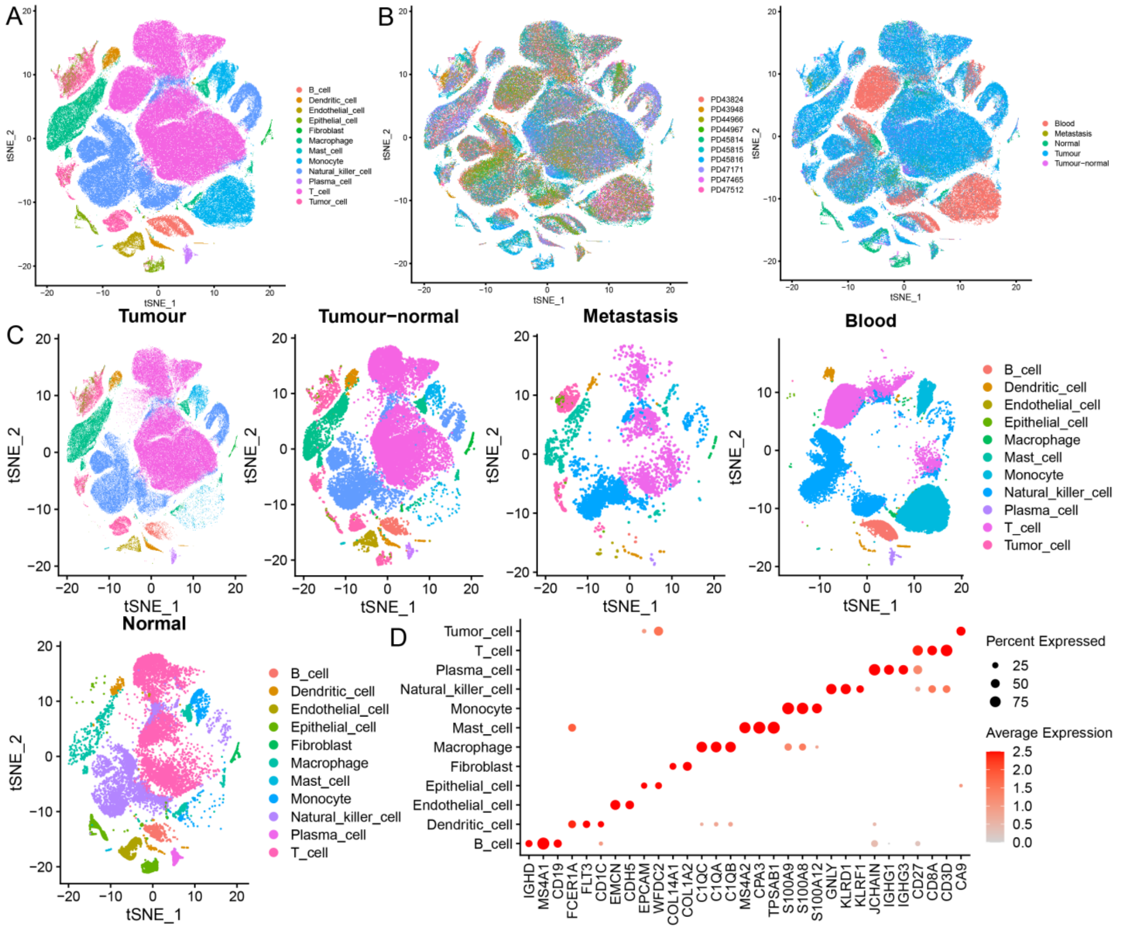

Figure S1: Multiregional single-cell transcriptome profiling of RCC. (A) TSNE of scRNA-seq showing eleven major cell types identified by marker gene expression. (B) TSNE showing classification of scRNA-seq data by 10 RCC patients (left) and regions (right). Area plot showing changes in eleven cell types at the five regions of RCC. (C) TSNE showing classification of scRNA-seq data from 5 regions by cell type. Dot colors refer to cell types. (D) Dot plots show the average expression of marker genes in major cell types. Colors refer to expression level. Dot sizes refer to the proportion of gene expression in cells.

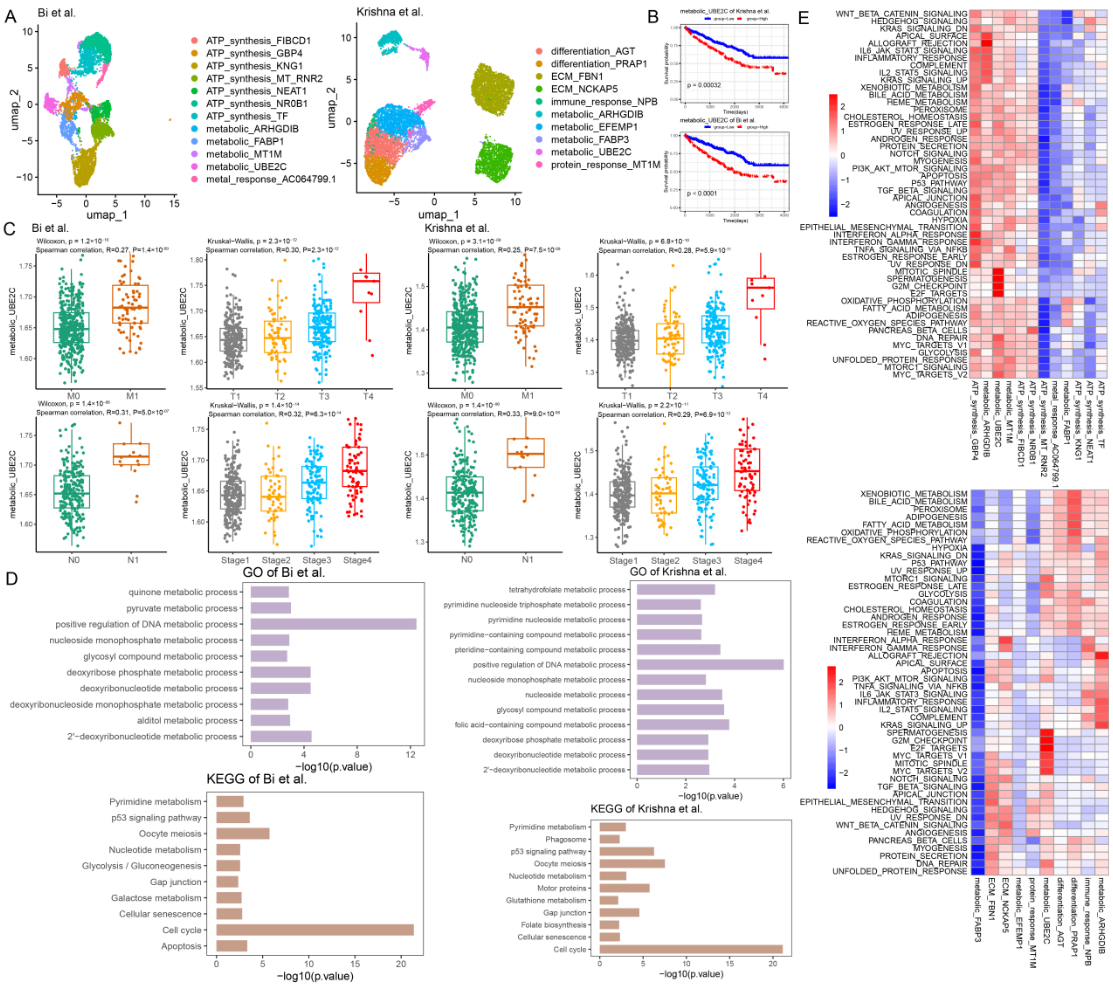

Figure S2: Cancer cell subpopulations of RCC tumorigenesis in two additional RCC scRNA-seq independent cohorts from Bi et al. and Krishna et al. (A) UMAP showing cancer cell subpopulations identified by marker gene expression in two additional RCC scRNA-seq independent cohorts. (B) The Kaplan-Meier survival curves of RCC patients stratified by metabolic^{high} UBE2C+ cancer cell infiltration. (C) In two additional RCC scRNA-seq independent cohorts, comparison of absolute infiltration proportion of metabolic^{high} UBE2C+ cancer cell in M classification, N classification, T classification, stage. (D) Pathway enrichment of differentially expressed genes in metabolic^{high} UBE2C+ cancer cell in two additional RCC scRNA-seq independent cohorts. (E) Heatmap shows the abundance of 50 hallmark genesets in the cancer cell subpopulation.

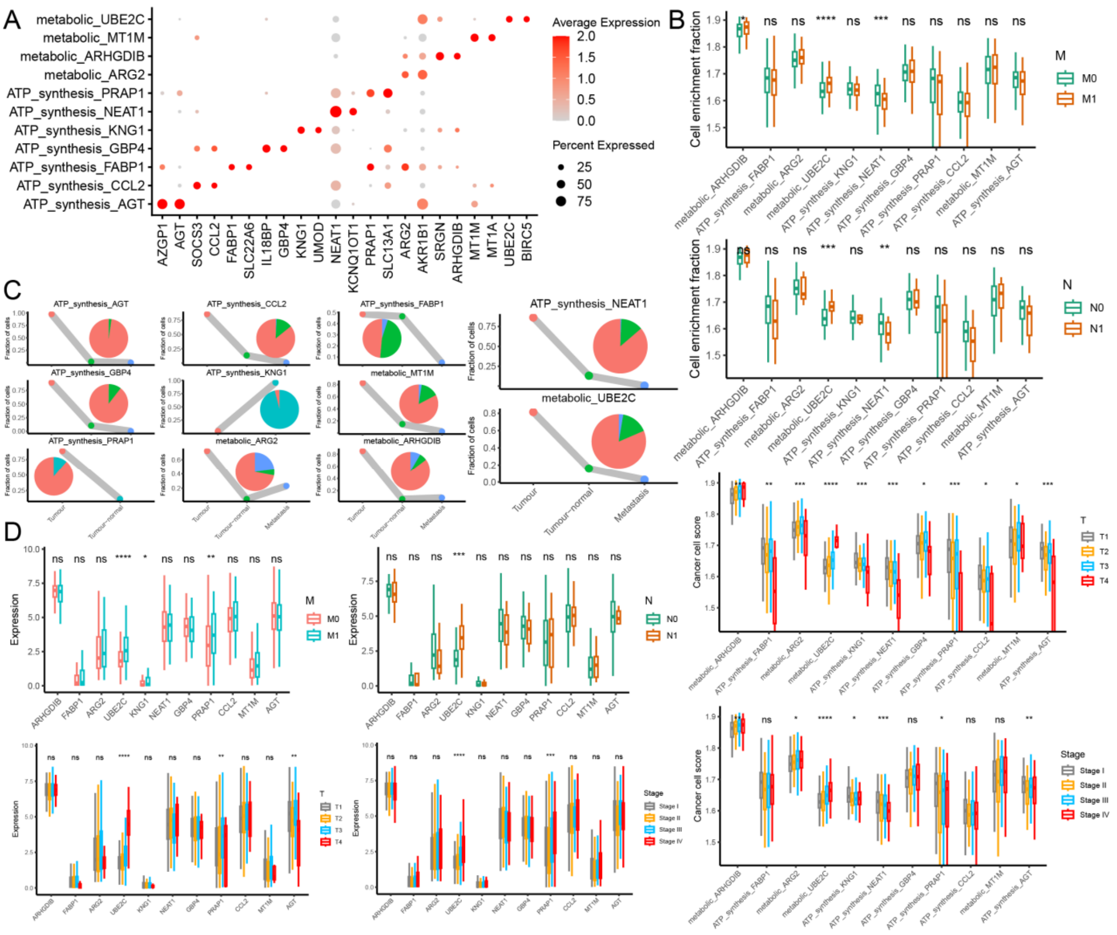

Figure S3: Cancer cell subpopulations of RCC tumorigenesis. (A) Dot plots show the average expression of marker genes in the cancer cell subpopulation. Colors refer to expression level. Dot sizes refer to the proportion of gene expression in cells. (B) Comparison of absolute infiltration proportion of tumor cell subpopulations in M classification, N classification, T classification, stage. (C) Area plot showing changes in cancer cell subpopulations among different disease regions of RCC. (D) Comparison of absolute infiltration proportion of marker genes in M classification, N classification, T classification, stage. The Kruskal-Wallis test and wilcoxon rank sum test were used for statistical significance. ****: P< 0.0001 ; ***: P< 0.001; **: P< 0.01; *: P< 0.05; ns: not significant.

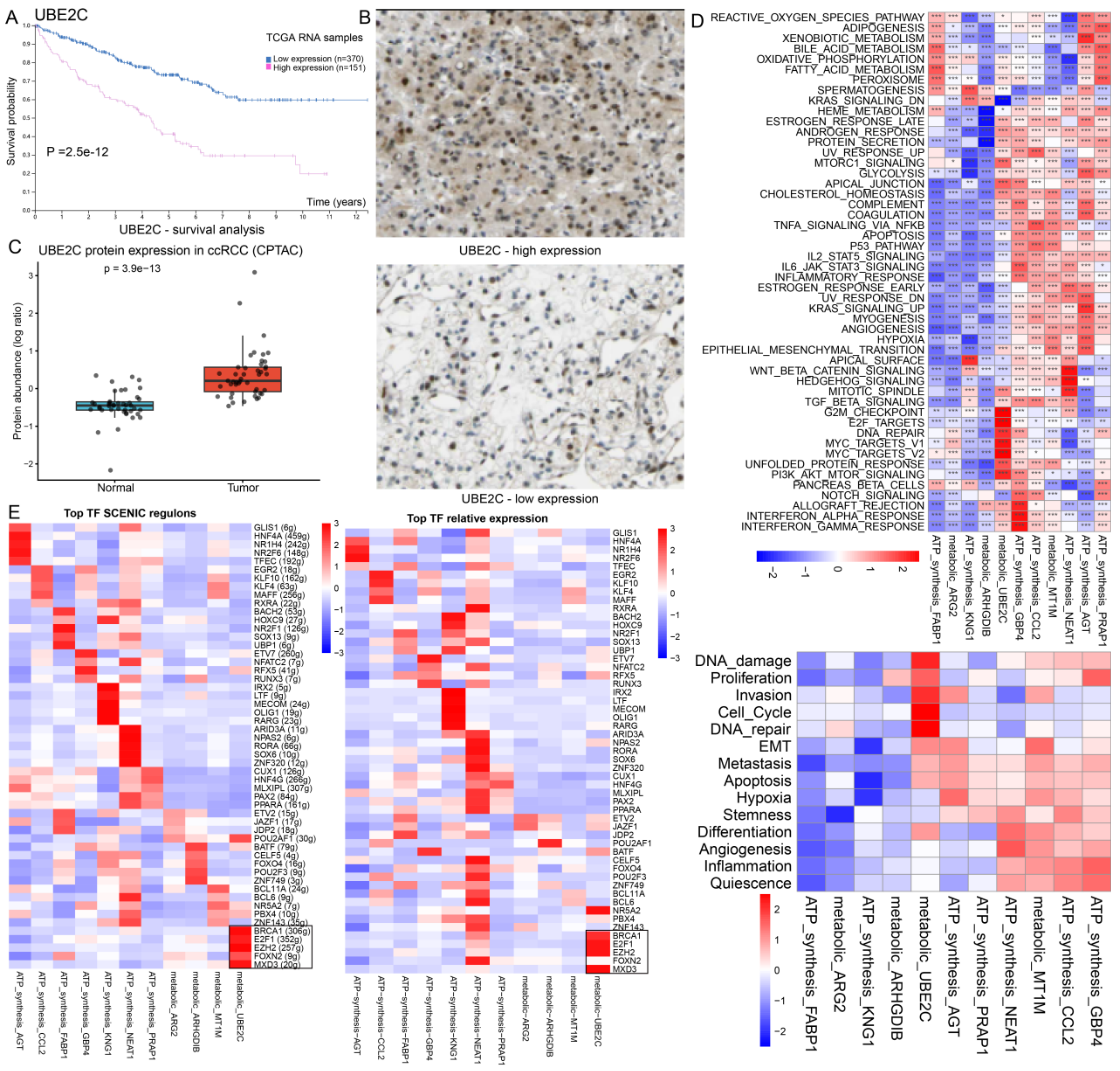

Figure S4: The marker gene UBE2C is significantly associated with the poor survival of patients. (A) overall survival (OS) based on UBE2C expression data of renal cancer. (B) lmmunohistochemical staining of UBE2C in renal cancer tissue, demonstrating a representative tissue sample classified as having a high level of protein expression and as having a low level of protein expression from The Human Protein Atlas (https://www.proteinatlas.org/). (C) UBE2C protein expression quantified in the CPTAC3 PDC000127 cohort. P-value was calculated by Wilcoxon rank-sum test. (D) Heatmap shows the abundance of 50 hallmark genesets in the cancer cell subpopulation and the abundance of 14 functional states in the cancer cell subpopulation. ***: P< 0.001; **: P< 0.01; *: P< 0.05. (E) Heatmap shows normalized activity of top 5 TF regulons in tumor cell subpopulations using pySCENIC (left) and the relative expression of top 5 TFs in each cancer cell subpopulations (right).

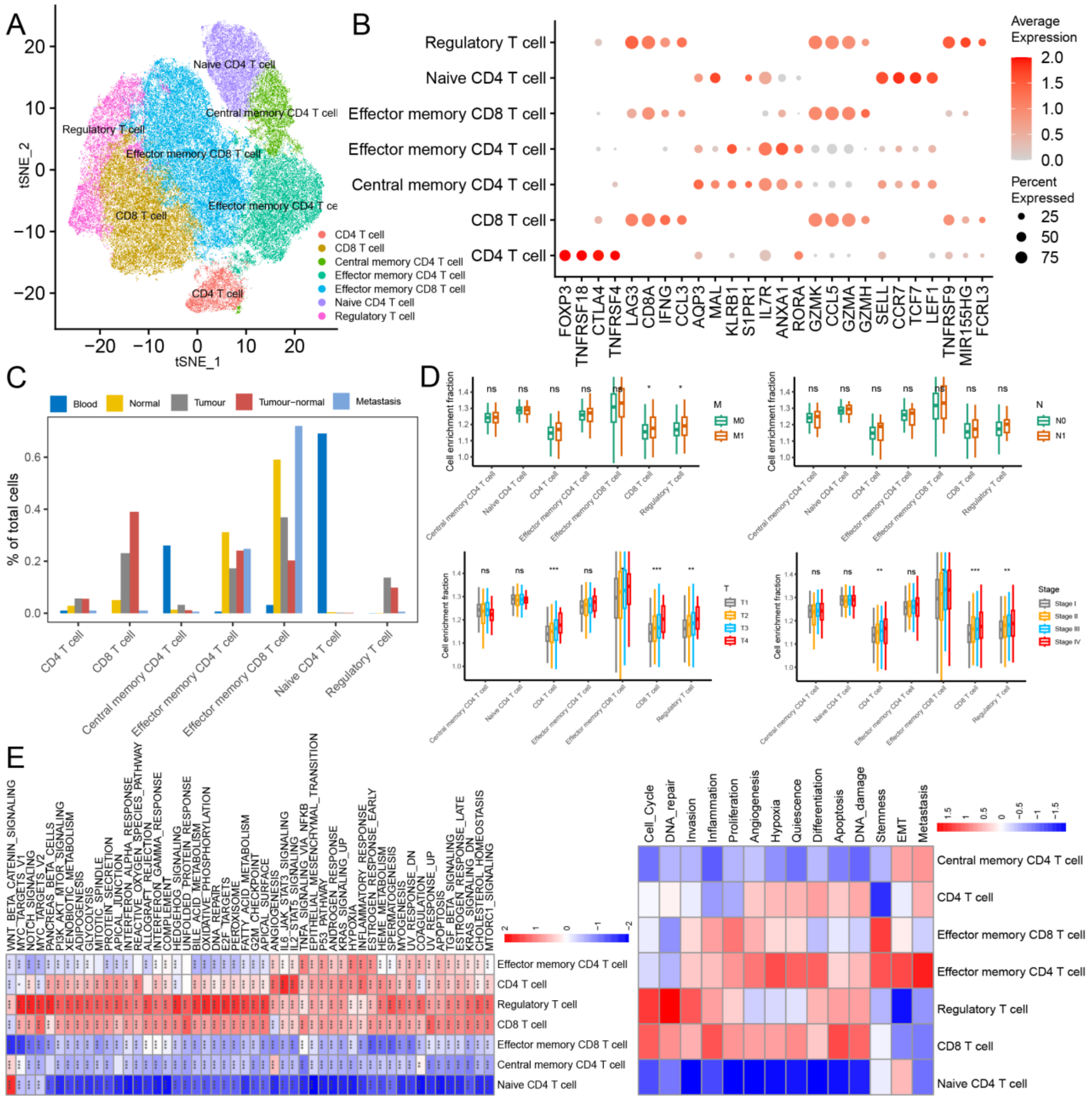

Figure S5: T-cell subpopulations of RCC tumorigenesis. (A) TSNE shows subpopulations of T-cells identified by marker gene expression. (B) Dot plots show the average expression of marker genes in T-cell subpopulation. Colors refer to expression level. Dot sizes refer to the proportion of gene expression in cells. (C) Bar plot shows the proportion of T-cell subpopulations enriched in different tumor regions. (D) Comparison of absolute infiltration proportion of T-cell subpopulations in M classification, N classification, T classification and stage. The Kruskal-Wallis test and wilcoxon rank sum test were used for statistical significance. ****: P< 0.0001 ; ***: P< 0.001; **: P< 0.01; *: P< 0.05; ns: not significant. (E) Heatmap shows the abundance of 50 hallmark genesets and 14 functional states in T cell subpopulation.

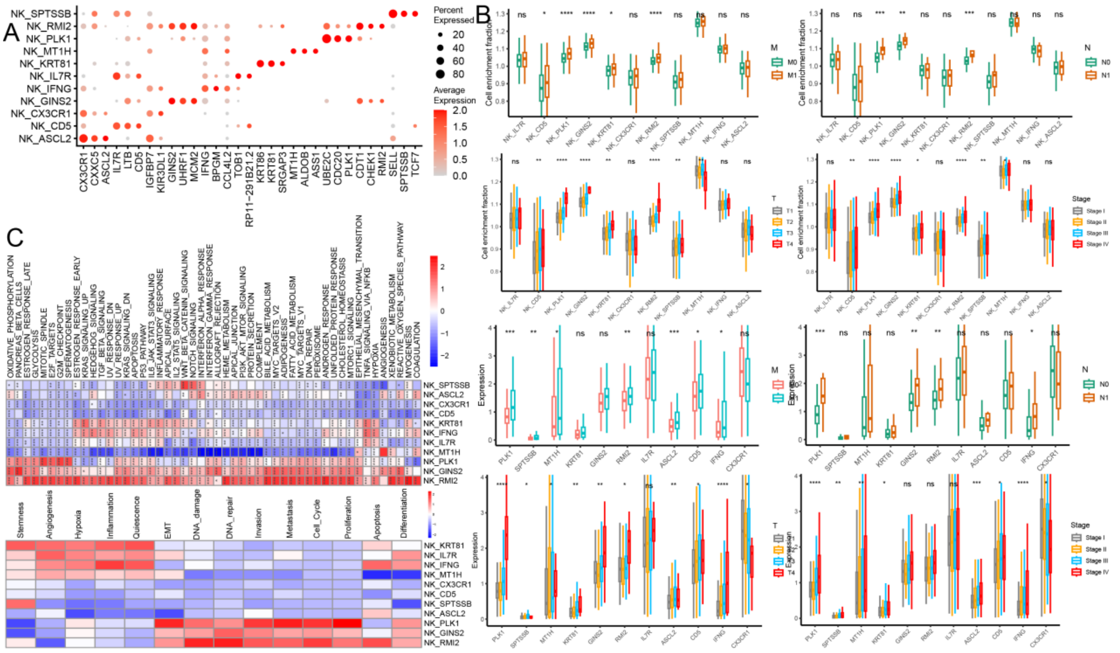

Figure S6. NK cell subpopulations of RCC tumorigenesis. (A) Dot plots show the average expression of marker genes in NK cell subpopulation. Colors refer to expression level. Dot sizes refer to the proportion of gene expression in cells. (B) Comparison of absolute infiltration proportion of NK cell subpopulation and marker genes in M classification, N classification, T classification and stage. The Kruskal-Wallis test and wilcoxon rank sum test were used for statistical significance. ****: P< 0.0001 ; ***: P< 0.001; **: P< 0.01; *: P< 0.05; ns: not significant. (C) Heatmap shows the abundance of 50 hallmark genesets and 14 functional states in NK cell subpopulation.

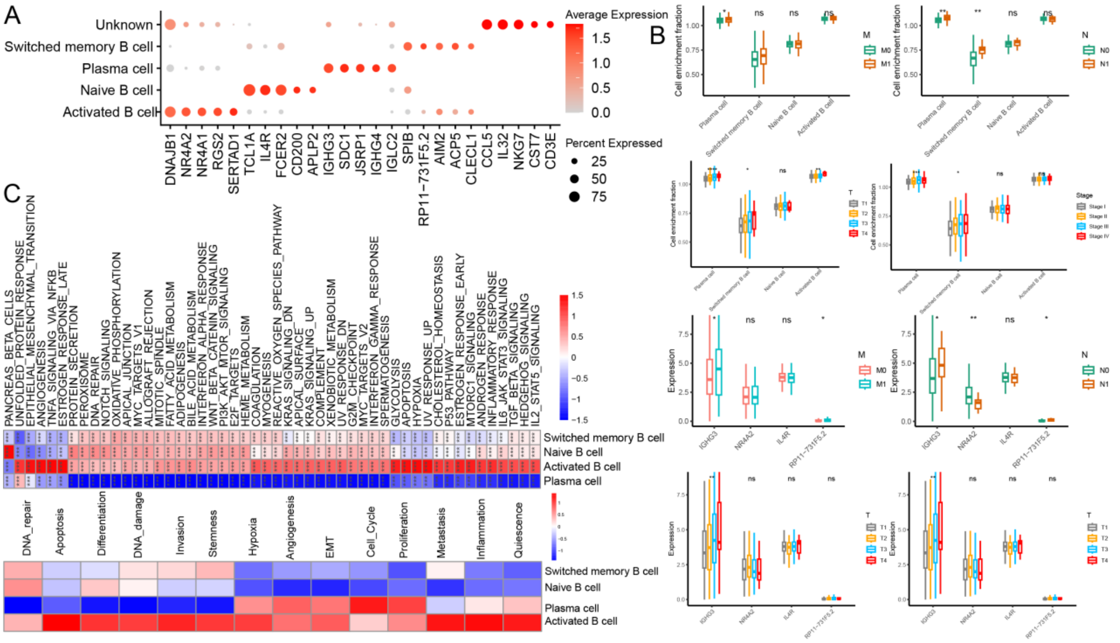

Figure S7: B-cell subpopulations of RCC tumorigenesis. (A) Dot plots show the average expression of marker genes in B-cell subpopulation. Colors refer to

expression level. Dot sizes refer to the proportion of gene expression in cells. (B) Comparison of absolute infiltration proportion of B-cell subpopulation and marker genes in M classification, N classification, T classification and stage. The Kruskal-Wallis test and wilcoxon rank sum test were used for statistical significance. ****: P< 0.0001 ; ***: P< 0.001; **: P< 0.01; *: P< 0.05. (C) Heatmap shows the abundance of 50 hallmark genesets and 14 functional states in B cell subpopulation. +

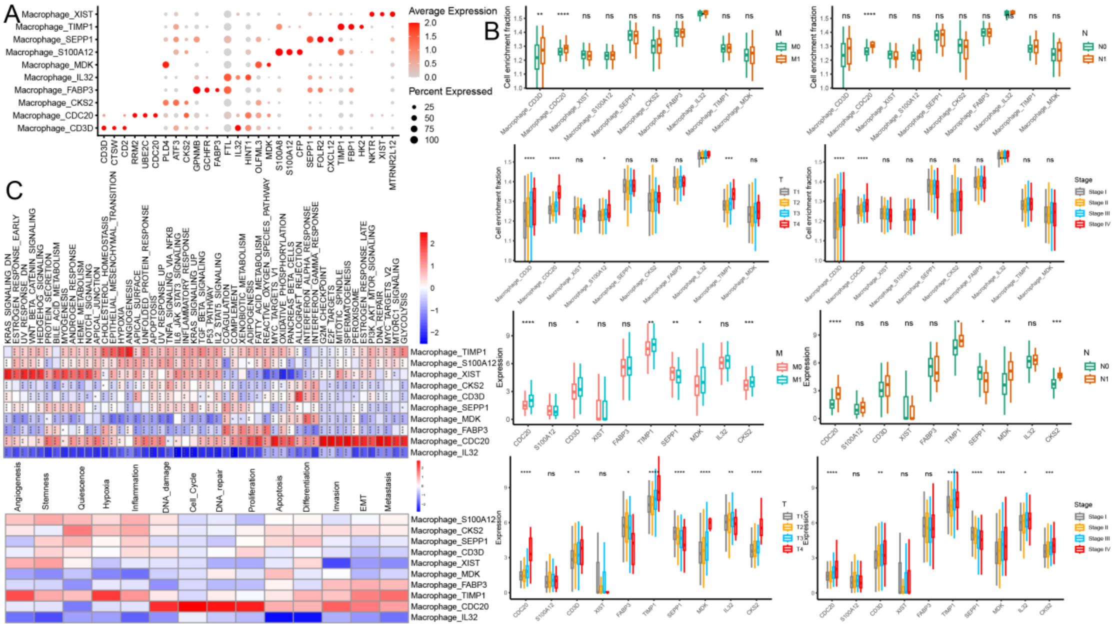

Figure S8: Macrophage subpopulations of RCC tumorigenesis. (A) Dot plots show the average expression of marker genes in macrophage subpopulations. Colors refer to expression level. Dot sizes refer to the proportion of gene expression in cells. (B) Comparison of absolute infiltration proportion of macrophage subpopulations and marker genes in M classification, N classification, T classification and stage. The Kruskal-Wallis test and wilcoxon rank sum test were used for statistical significance. ****: P< 0.0001 ; ***: P< 0.001; **: P< 0.01; *: P< 0.05; ns: not significant. (C) Heatmap shows the abundance of 50 hallmark genesets and 14 functional states in macrophage subpopulations.

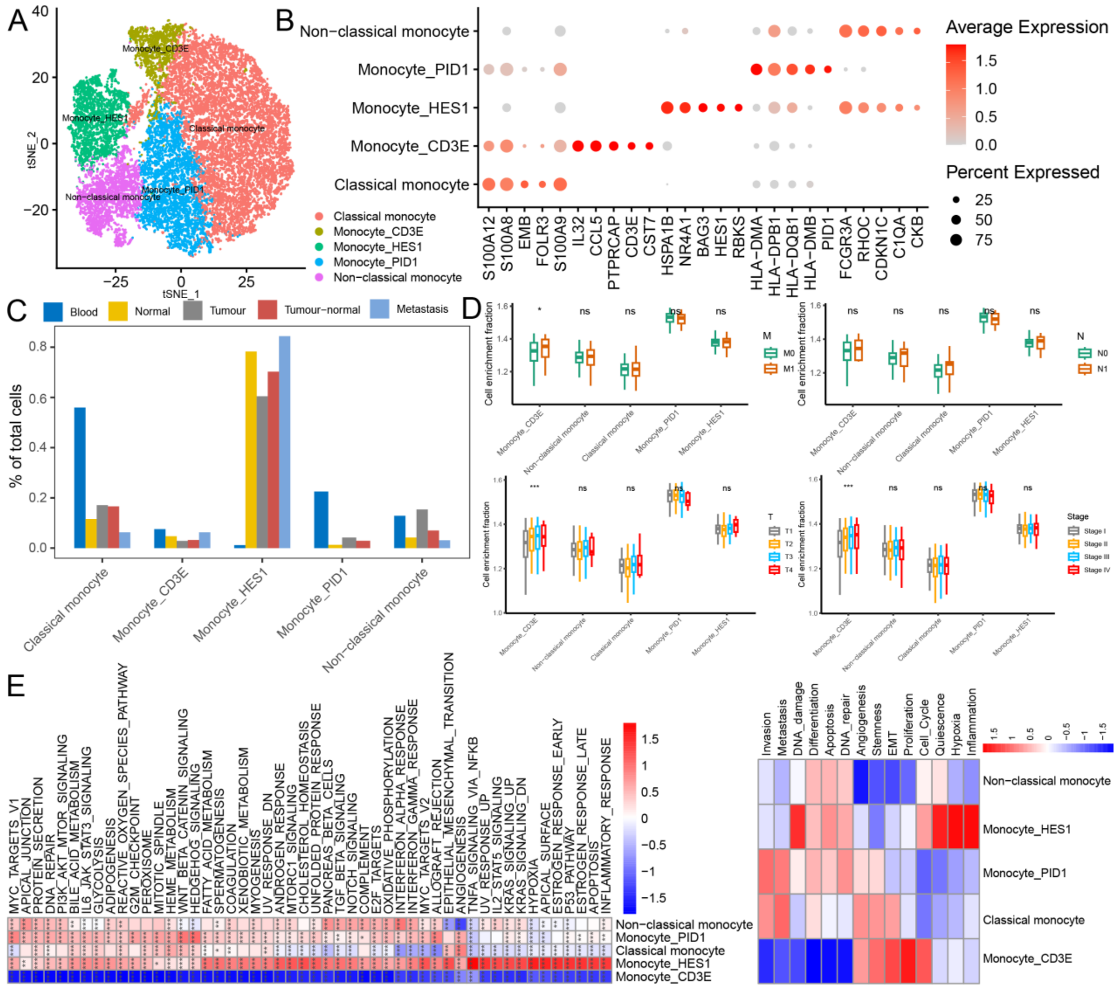

Figure S9: Monocyte subpopulations of RCC tumorigenesis. (A) TSNE shows subpopulations of monocytes identified by marker gene expression. (B) Dot plots show the average expression of marker genes in monocyte subpopulation. Colors refer to expression level. Dot sizes refer to the proportion of gene expression in cells. (C) Bar plot shows the proportion of monocyte subpopulations enriched in different tumor regions. (D) Comparison of absolute infiltration proportion of monocyte subpopulations in M classification, N classification, T classification and stage. The Kruskal-Wallis test and wilcoxon rank sum test were used for statistical significance. ****: P< 0.0001 ; ***: P< 0.001; **: P< 0.01; *: P< 0.05; ns: not significant. (E) Heatmap shows the abundance of 50 hallmark genesets and 14 functional states in monocyte subpopulations.

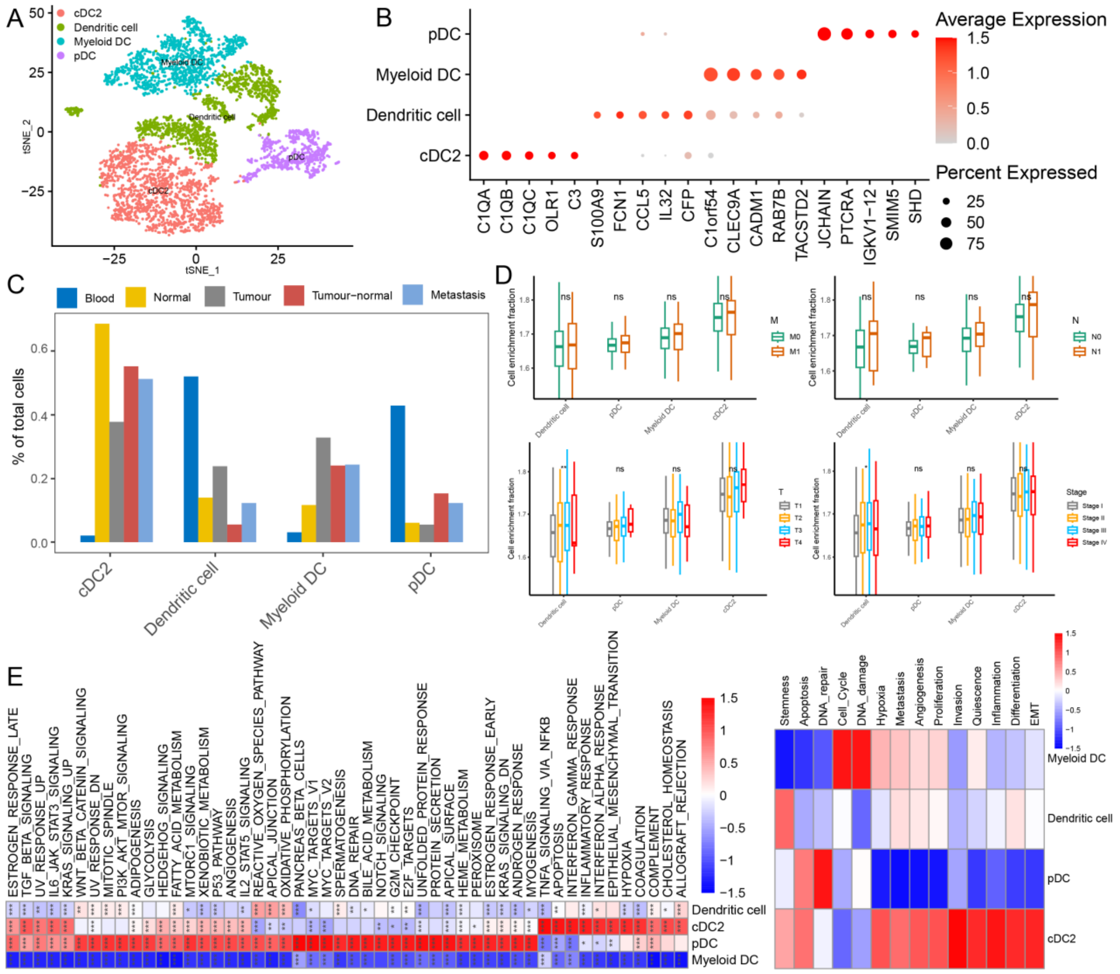

Figure S10: Dendritic cell subpopulations of RCC tumorigenesis. (A) TSNE shows subpopulations of dendritic cells identified by marker gene expression. (B) Dot plots show the average expression of marker genes in dendritic cell subpopulation. Colors refer to expression level. Dot sizes refer to the proportion of gene expression in cells. (C) Bar plot shows the proportion of dendritic cell subpopulations enriched in different tumor regions. (D) Comparison of absolute infiltration proportion of dendritic cell subpopulations in M classification, N classification, T classification and stage. The Kruskal-Wallis test and wilcoxon rank sum test were used for statistical significance. ****: P< 0.0001 ; ***: P< 0.001; **: P< 0.01; *: P< 0.05; ns: not significant. (E) Heatmap shows the abundance of 50 hallmark genesets and 14 functional states in dendritic cell subpopulations.

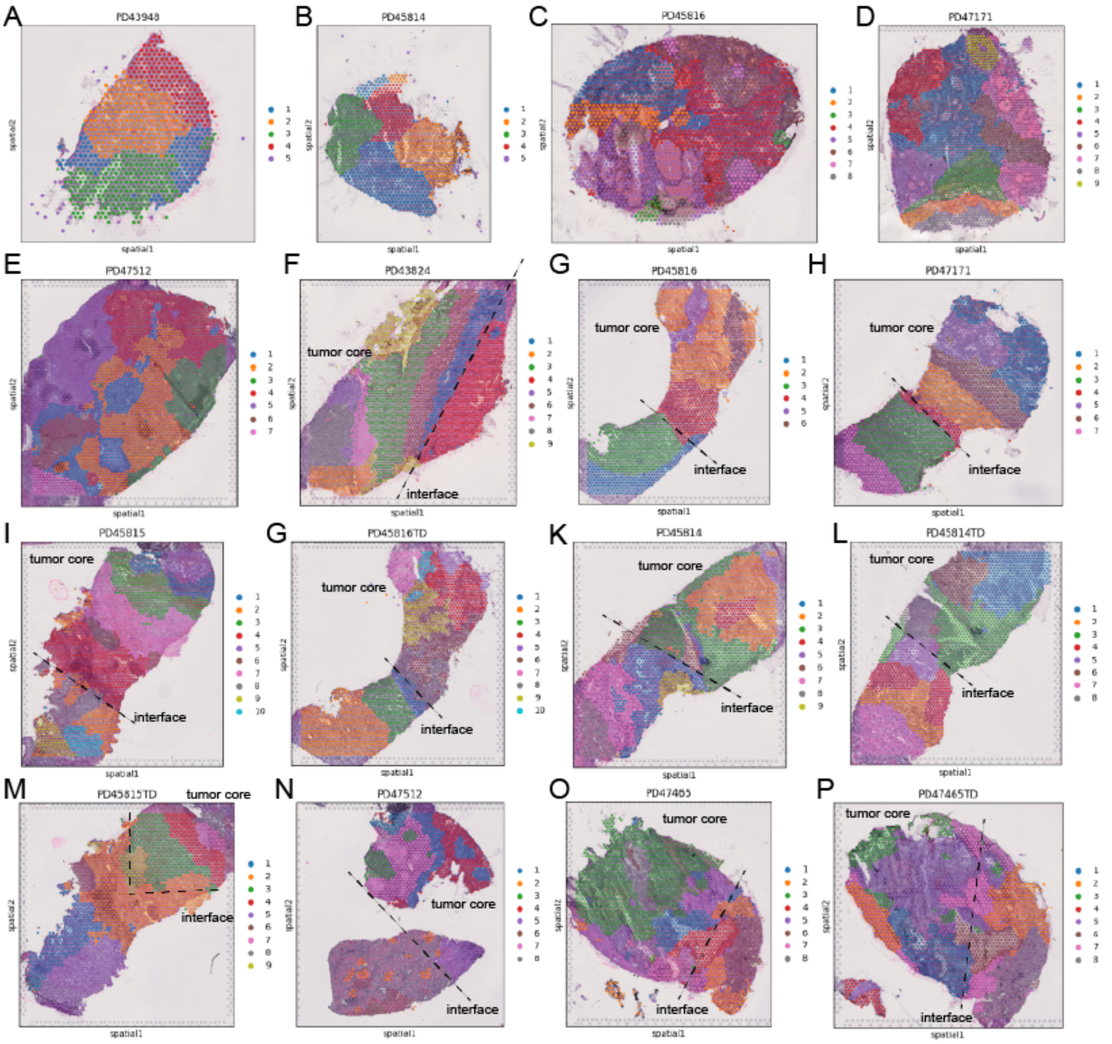

Figure S11: Presentation of spatial transcriptomic data. (A-E) Clustering of ST points by STAGATE algorithm in tumor core tissue sections. (F-P) Clustering of ST points by STAGATE algorithm in tumor-normal interface tissue sections. Dot colors refer to cluster of ST.

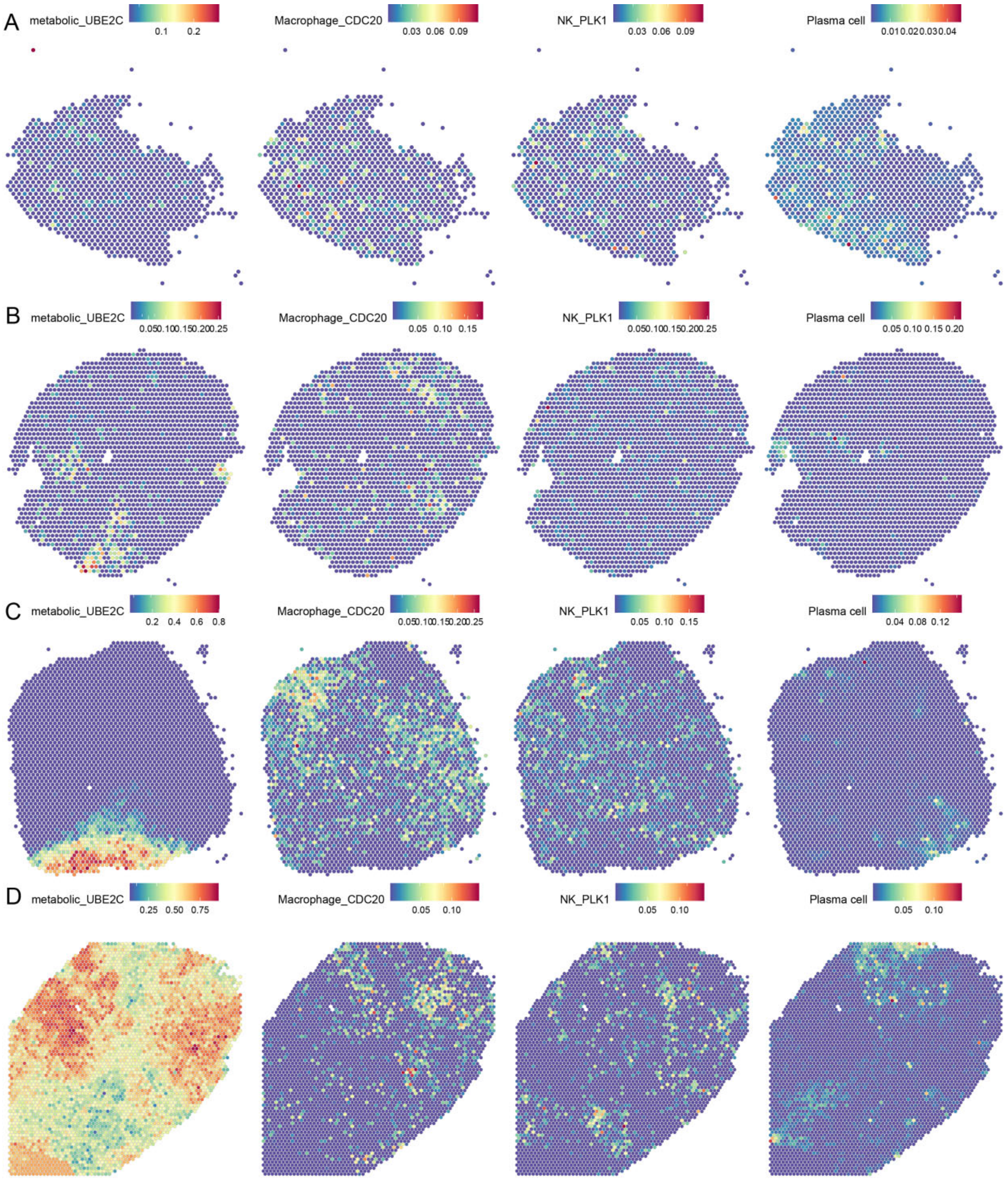

Figure S12: Spatial feature plots of signature score of metabolic^{high} UBE2C+ cancer cells, and risk TME immune cell subpopulations in tumor core tissue sections, including PD45814 (A), PD45816 (B), PD47171 (C) and PD47512 (D). Colors refer to cell abundance.

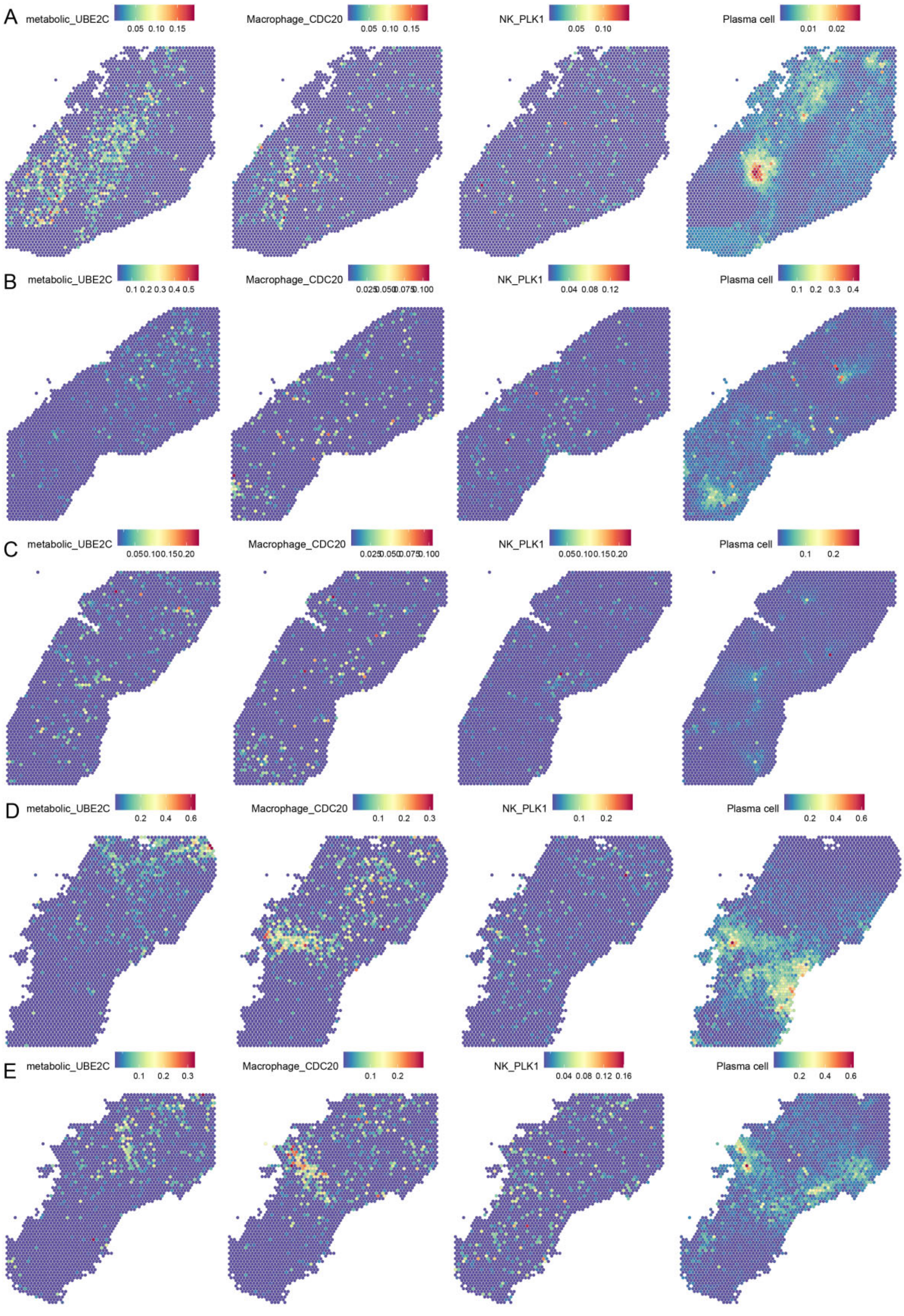

Figure S13: Spatial feature plots of signature score of metabolic^{high} UBE2C+ cancer cells, and risk TME immune cell subpopulations in tumor-normal interface tissue sections, including PD43824 (A), PD45814 (B), PD45814TD (C), PD45815 (D) and PD45815TD (E). Colors refer to cell abundance.

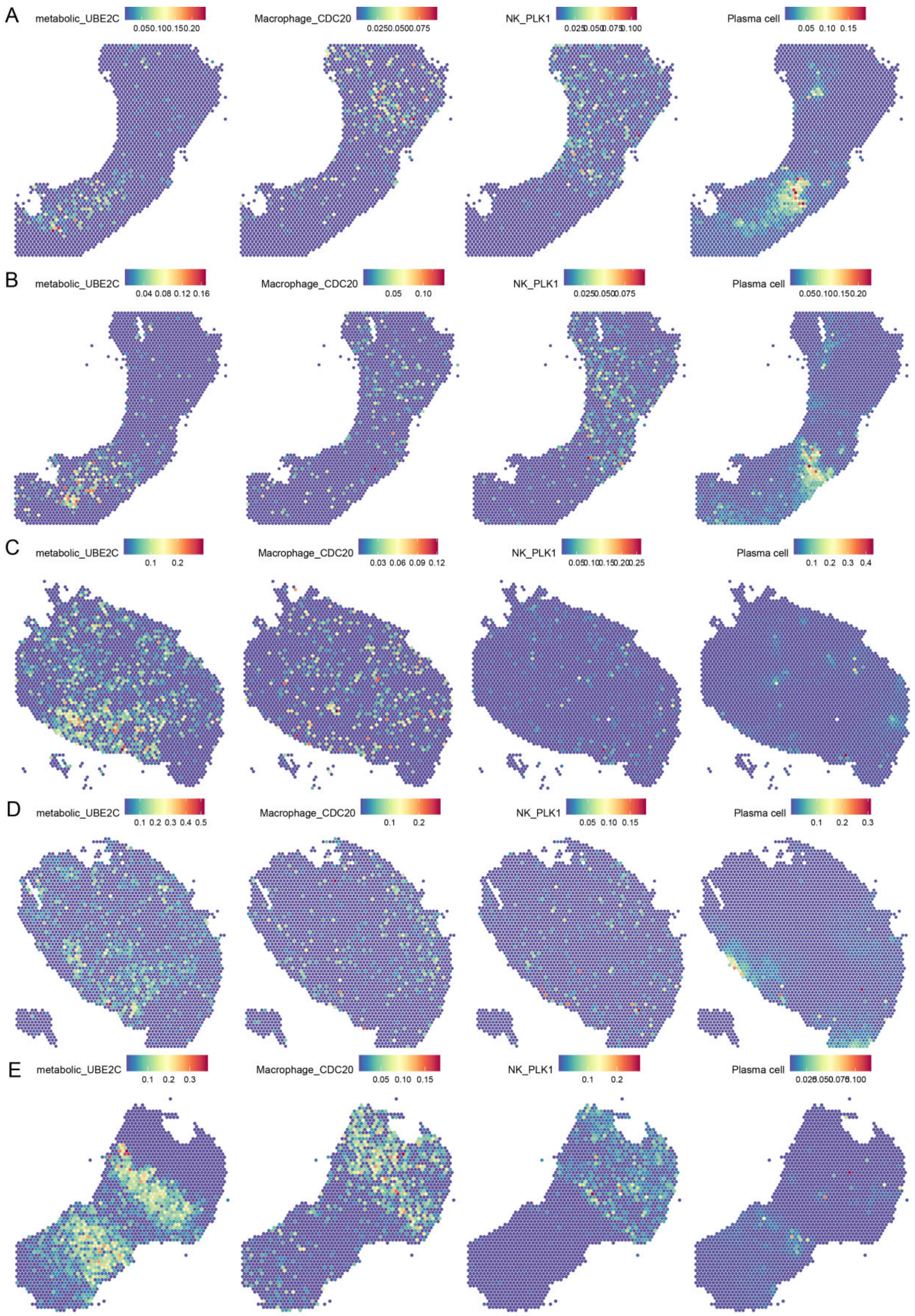

Figure S14: Spatial feature plots of signature score of metabolic^{high} UBE2C+ cancer cells, and risk TME immune cell subpopulations in tumor-normal interface tissue sections, including PD45816 (A), PD45816TD (B), PD47465 (C), PD47465TD (D) and PD47171 (E). Colors refer to cell abundance.

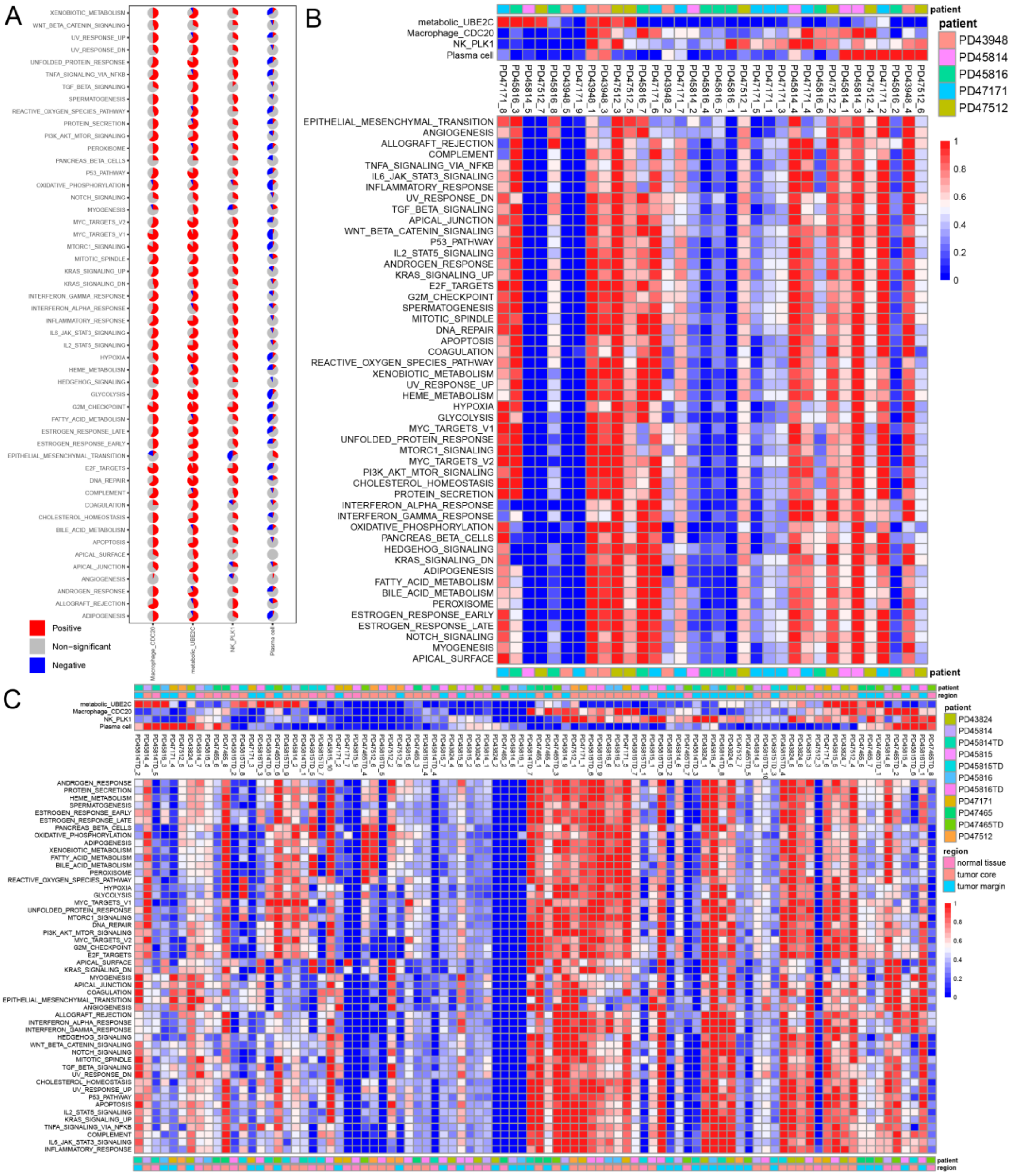

Figure S15: Functional distribution of spatial region. (A) The correlation of signature score of 50 hallmark gene sets and cell subpopulations in the tumor core sections and tumor-normal interface sections. Positive correlation is red, negative is blue, non-significant correlation is gray. (B) Heatmap shows the signature score of 50 hallmark gene sets, cancer cells and immune cells in each cluster in the tumor core sections. (C) Heatmap shows the signature score of 50 hallmark gene sets, cancer cells and immune cells in each cluster in the tumor-normal interface sections.

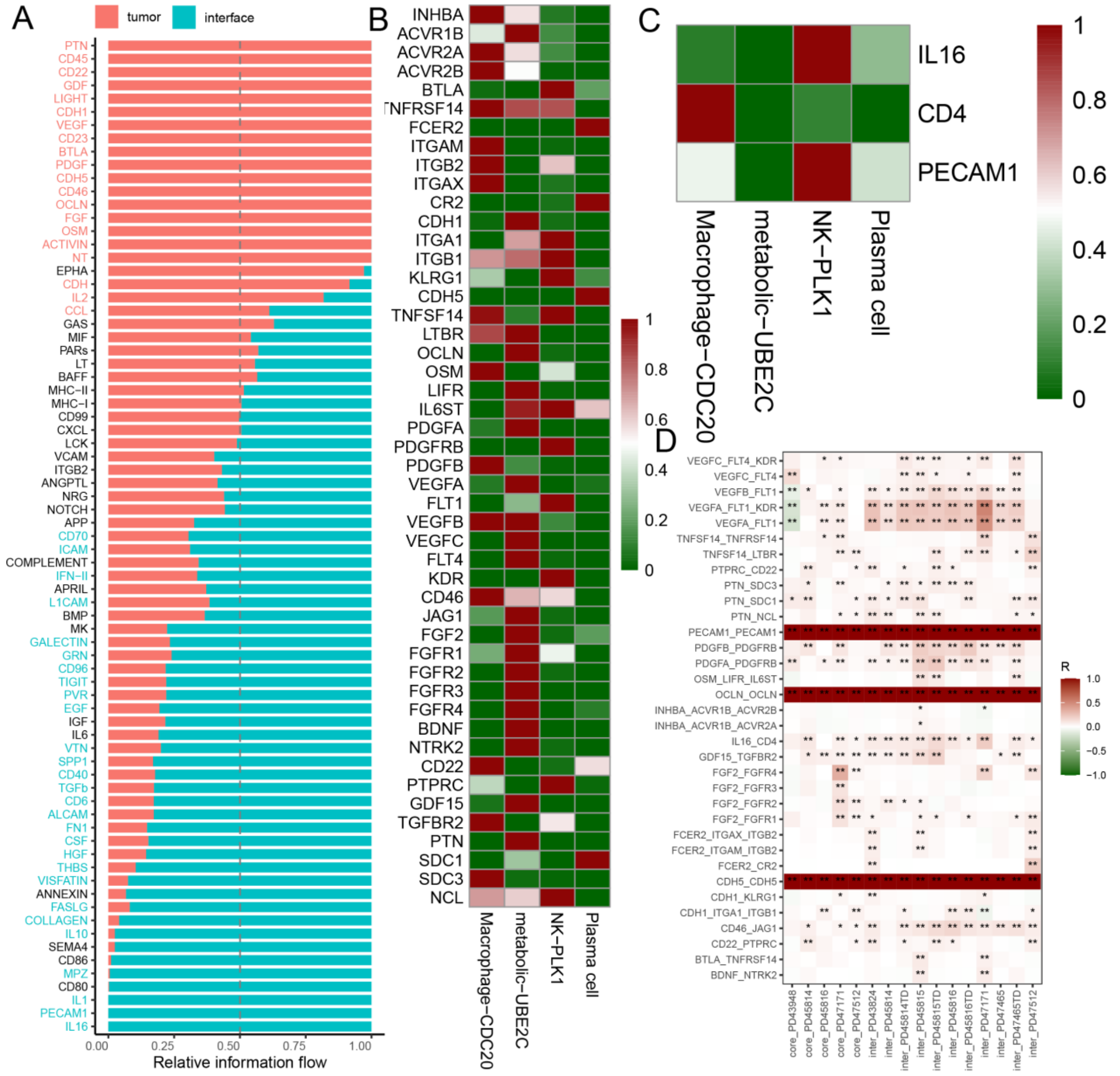

Figure S16: Analysis of cell communication between the tumor core and tumor-normal interface. (A) Bar plot displayed specific enrichment of signaling pathways between cancer cells and risk immune cells of tumor core and tumor-normal interface. (B-C) Heatmap displayed the expression levels of specific receptors and ligands in cancer cells and risk immune cells in tumor core (B) and tumor-normal interface (C). Colors refer to expression level. (D) The Spearman correlation of ligand-receptor pairs in 16 spatial transcriptomics sections. Colors refer to correlation level.

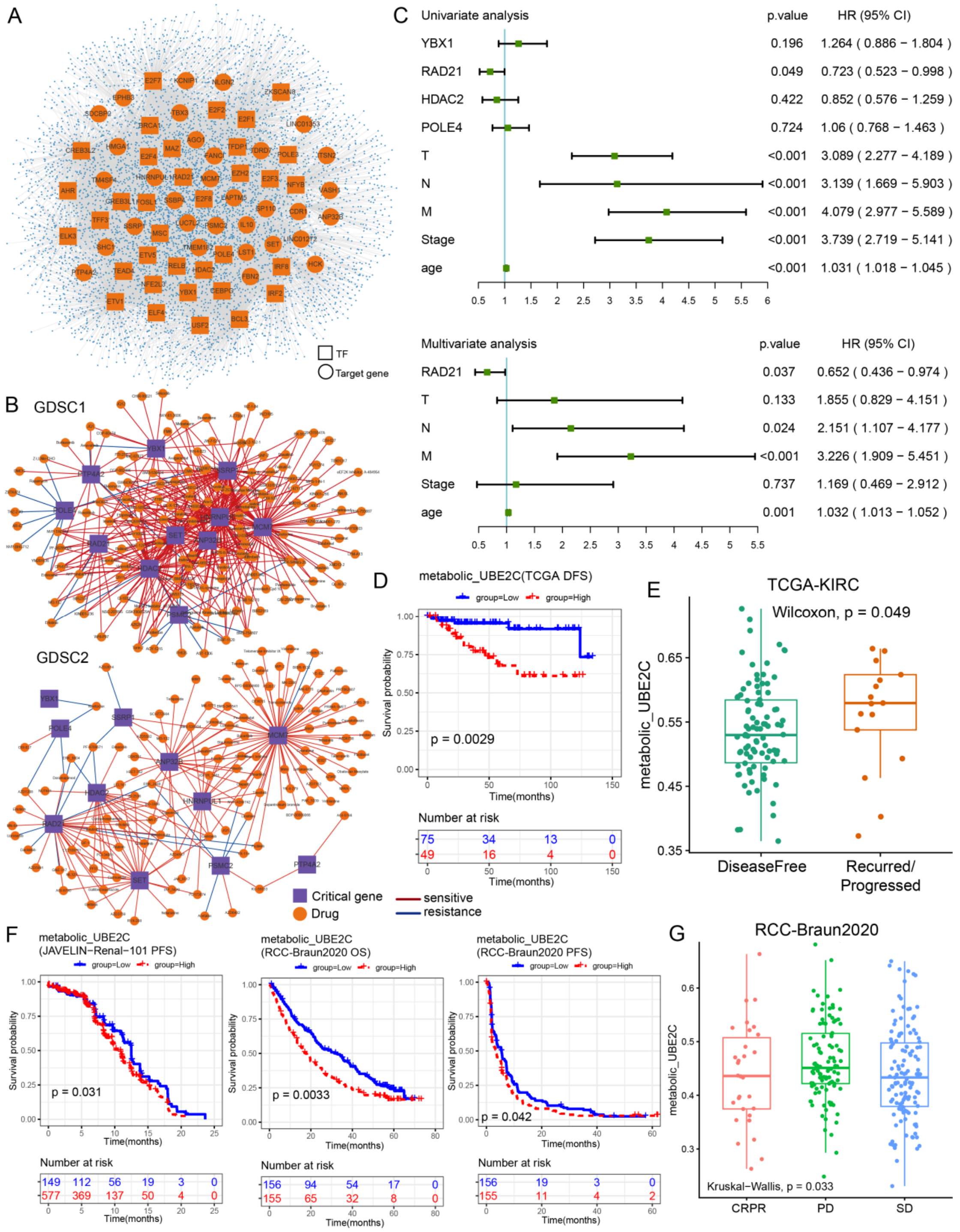

Figure S17: Potential drugs targeting critical TFs. (A) GRNs of metabolic^{high} UBE2C+ cancer cells. Dot shapes refer to TF or target gene. (B) The network of critical genes and critical gene-related drugs. Line colors refer to sensitive or resistant. Dot shapes refer to TF or drug. (C) Univariate Cox regression analysis and multivariate Cox regression analysis validated critical TFs as an independent prognostic factor in TCGA cohort. (D) Survival analyses for low and high infiltration of metabolic^{high} UBE2C+ cancer cells patient groups in the TCGA-KIRC cohort (disease-free survival, DFS). (E) The difference in the abundance of metabolic^{high} UBE2C+ cancer cells between disease recurrence and non-recurrence. (F) Survival

analyses for low and high infiltration of metabolic^{high} UBE2C+ cancer cells patient groups in the JAVELIN-Renal-101 (progression-free survival, PFS) and RCC-Braun2020 (PFS and OS) cohorts. (G) The difference in the abundance of metabolic^{high} UBE2C+ cancer cells among patients with different treatment responses in RCC-Braun2020 cohort.

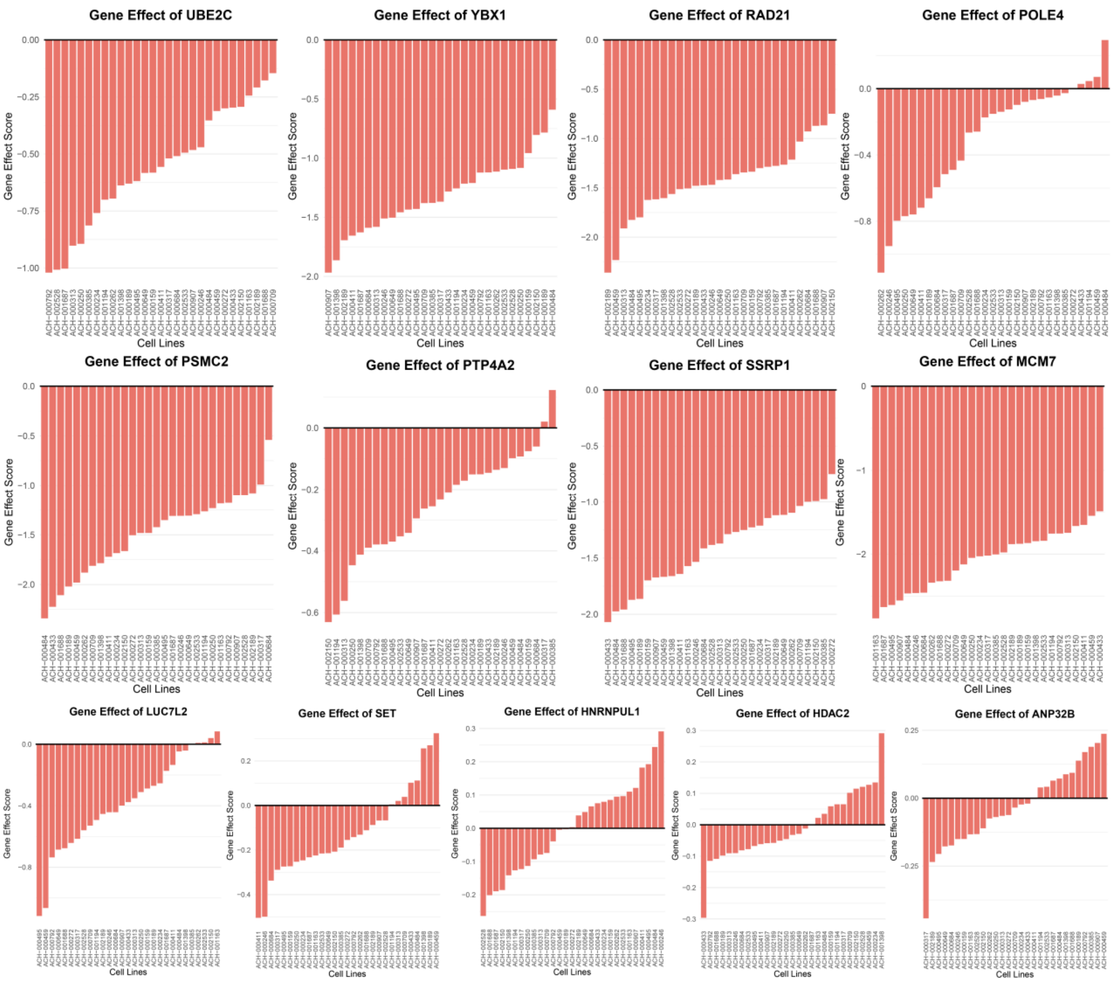

Figure S18: The functional impact of critical genes knockdown in genome-wide CRISPR-Cas9 screening data.

Table S1: Composition of recurrent gene modules

Module	Genes
Metal response	AC090498.1, ACTB, B2M, FTL, GSTP1, HSPB1, NNMT, RPLP1, RPS12, S100A11
ATP synthesis	MT-CO1, MT-CO3, MT-CO2, MT-ND4L, MT-ATP8, MT-CYB, MT-ND3, MT-ATP6, MT-ND4, MT-ND1
metabolic	ENO1, GAPDH, HLA-A, HLA-B, LDHA, NDUFA4L2, PKM, RPL7, S100A11, TPI1, VIM
Immune response	HLA-B, HLA-A, GAPDH, HLA-C, VIM, CD74, IGFBP3, HLA-DRB1, HLA-DRA, CYBA, CD63, TPI1, ANGPTL4, C1S, C1R, NDUFA4L2
differentiation	ACTB, ACTG1, CD74, CLU, CYB5A, FXYD2, GPX3, IFITM3,

	MTRNR2L12, NNMT, SERPINA1, SPP1, TMEM176A, TMEM176B
ECM	MTRNR2L8, VCAN, ANXA2, CAV1, DDIT4, TAGLN2, LGALS1, ACTG1, MTRNR2L12, ACTB, SPARC, SH3BGRL3, PFN1, WWTR1, TUBB, CD99, MT2A, S100A16
Protein response	HSPA1A, DNAJB1, FOS, HSPA1B, IFITM3, DUSP1, MT2A, HSP90AA1, JUN, FOSB, DDIT4, ACTG1, TAGLN2, NNMT, ACTB, ZFP36, CD74, LGALS1, SOD2, CLU
Stress response	FOS, EGR1, JUNB, ATF3, IER2, JUN, ZFP36, CDKN1A, MALAT1, ACTB, GADD45B, CRYAB, PPP1R15A, DUSP1, FOSB, RHOB, KLF6, ZFP36L1

Table S2: Statistical information table

Result section	Condition	Statistics
Result 2.2	Figure 2C	log-rank test: $P < 1.0 \times 10^{-4}$
	Figure 2D	M (Wilcoxon: $P=5.7 \times 10^{-6}$; Spearman: R=0.25, $P=2.1 \times 10^{-8}$)<br>T (Kruskal-Wallis: $P=1.4 \times 10^{-10}$; Spearman: R=0.28, $P=6.4 \times 10^{-11}$)<br>N (Wilcoxon: $P=2.1 \times 10^{-4}$; Spearman: R=0.25, $P=3.9 \times 10^{-5}$)<br>Stage (Kruskal-Wallis: $P=3.0 \times 10^{-10}$; Spearman: R=0.31, $P=6.4 \times 10^{-13}$)
	Figure S2B	Bi et al.(log-rank test: $P = 3.2 \times 10^{-3}$)<br>Krishna et al.(log-rank test: $P < 1.0 \times 10^{-4}$)
	Figure S2C	Bi et al.<br>M (Wilcoxon: $P=1.2 \times 10^{-10}$; Spearman: R=0.27, $P=1.4 \times 10^{-9}$)<br>T (Kruskal-Wallis: $P=2.3 \times 10^{-12}$; Spearman: R=0.30, $P=2.3 \times 10^{-12}$)<br>N (Wilcoxon: $P=1.4 \times 10^{-6}$; Spearman: R=0.31, $P=5.0 \times 10^{-7}$)<br>Stage(Kruskal-Wallis:$P=1.4 \times 10^{-14}$; Spearman: R=0.32, $P=6.3 \times 10^{-14}$)<br>Krishna et al.<br>M (Wilcoxon: $P=3.1 \times 10^{-9}$; Spearman: R=0.25, $P=2.1 \times 10^{-8}$)<br>T (Kruskal-Wallis: $P=1.4 \times 10^{-10}$; Spearman: R=0.28, $P=5.9 \times 10^{-11}$)<br>N (Wilcoxon: $P=1.4 \times 10^{-6}$; Spearman: R=0.33, $P=9.0 \times 10^{-8}$)<br>Stage(Kruskal-Wallis:$P=2.2 \times 10^{-11}$; Spearman: R=0.29, $P=6.9 \times 10^{-12}$)
	Figure S4A	log-rank test: $P = 2.5 \times 10^{-12}$
	Figure S4C	log-rank test: $P = 3.9 \times 10^{-13}$
Result 2.3	Figure 3C	NK_PKL1(log-rank test: $P < 6.0 \times 10^{-4}$)<br>PKL1(log-rank test: $P = 2.6 \times 10^{-4}$)<br>Macrophage_CDC20(log-rank test: $P = 3.3 \times 10^{-3}$)<br>CDC20(log-rank test: $P = 2.1 \times 10^{-4}$)<br>Plasma_cell(log-rank test: $P = 6.3 \times 10^{-4}$)<br>IGHG3(log-rank test: $P < 1.0 \times 10^{-4}$)
	Figure 3D	NK_PKL1:<br>M(Wilcoxon: $P=5.9 \times 10^{-6}$; Spearman: R=0.20, $P=4.8 \times 10^{-6}$)<br>N(Wilcoxon: $P=3.3 \times 10^{-4}$; Spearman: R=0.23, $P=2.8 \times 10^{-4}$)<br>T(Kruskal-Wallis: $P=1.7 \times 10^{-10}$; Spearman: R=0.27,$P=1.3 \times 10^{-10}$)<br>Stage(Kruskal-Wallis: $P=4.0 \times 10^{-9}$;Spearman: R=0.27,$P=2.8 \times 10^{-10}$)<br>Macrophage_CDC20:<br>M(Wilcoxon: $P=9.6 \times 10^{-6}$; Spearman: R=0.20, $P=8.0 \times 10^{-6}$)

		N(Wilcoxon: P=4.8×10^{-5}; Spearman: R=0.26, P=3.7×10^{-5})<br>T(Kruskal-Wallis: P=1.9×10^{-9};Spearman: R=0.15,P=9.5×10^{-9})<br>Stage(Kruskal-Wallis: P=2.6×10^{-8}; Spearman:R=0.24, P=1.5×10^{-8})<br>Plasma_cell:<br>M(Wilcoxon: P=1.0×10^{-2}; Spearman: R=0.12, P=1.0×10^{-2})<br>N(Wilcoxon: P=3.6×10^{-3}; Spearman: R=0.18, P=3.4×10^{-3})<br>T(Kruskal-Wallis: P=1.9×10^{-9}; Spearman: R=0.20,P=2.8×10^{-6})<br>Stage(Kruskal-Wallis: P=2.6×10^{-8}; Spearman:R=0.19, P=1.2×10^{-5})
	PDCD1	log2FC=2.6, FDR<0.05
	HAVCR2	log2FC=0.66, FDR=72×10^{-105}
	LAG3	log2FC=3.4, FDR<0.05
	FCGR3A	log2FC=-3.7, FDR<0.05
	NCR3	log2FC=-2.6, FDR=6.4×10^{-269}
	PRDM1	log2FC=3.8, FDR=3.5×10^{-290}
	PIM2	log2FC=2.5, FDR=8.4×10^{-279}
	CREB3L2	log2FC=2.8, FDR=1.7×10^{-60}
	CCL4	log2FC=0.87, FDR=4.4×10^{-7}
	FN1	log2FC=0.28, FDR=4.7×10^{-9}
Result 2.4	Figure 4D	PD43948: macrophage_CDC20-metabolic_UBE2C (Spearman: R=0.38, P<2.2×10^{-16})<br>PD43948: NK_PKL1-metabolic_UBE2C (Spearman: R=0.38, P<2.2×10^{-16})<br>PD47512: macrophage_CDC20-metabolic_UBE2C (Spearman: R=0.66, P<2.2×10^{-16})<br>PD47512: NK_PKL1-metabolic_UBE2C (Spearman: R=0.6, P<2.2×10^{-16})<br>PD43948: Macrophage_CDC20-NK_PKL1 (Spearman: R=0.66, P< 2.2×10^{-16})<br>PD47512: macrophage_CDC20-NK_PKL1 (Spearman: R=0.56, P< 2.2×10^{-16})
Result 2.6	Figure S17D	log-rank test: P =4.9×10^{-2}
	Figure S17E	Wilcoxon: P=3.1×10^{-9}
	Figure S17F	JAVELIN-Renal-101 PFS(log-rank test: P =3.1×10^{-2})<br>RCC-Braun2020 OS(log-rank test: P =3.3×10^{-3})<br>RCC-Braun2020 PFS(log-rank test: P =4.2×10^{-2})
	Figure S17G	Kruskal-Wallis: P=3.3×10^{-2}

Table S3: List of scRNA datasets.

Data source	Cancer	Cells	PMID	Patient characteristics
GSE176078	Breast Cancer	91014	34493872	Number of patients: 26<br>Age: 54(49-61)<br>Sex: Male(0)/Female(26)<br>Grade: II(9)/III(17)
GSE225857	Colorectal Cancer	208589	37327339	Number of patients: 6<br>Age: 61(58-72)<br>Sex: Male(4)/Female(2)
GSE169379	Muscle Invasive Bladder Cancer	67988	34385456	--
GSE156625	Liver Hepatocellular Carcinoma	58831	32976798	Number of patients: 14<br>Age: 69(56-74)<br>Sex: Male(11)/Female(3)
GSE189357	Lung Adenocarcinoma	122373	36434043	Number of patients: 9<br>Age: 56(45-57)<br>Sex: Male(3)/Female(6)<br>Histological type: AIS(3)/MIA(3)/IAC(3)
GSE202742	Pancreatic Adenocarcinoma	16648	37524695	Number of patients: 4
GSE185386	Skin Cutaneous Melanoma	29497	35803246	Number of patients: 5
https://doi.org/10.17632/g67bkbnhhg.1 (Li et al.)	Renal Cell Carcinoma	243864	36423636	Number of patients: 10<br>Age: 60(55-72.5)<br>Sex: Male(6)/Female(4)<br>Stage: I(2)/III(7)

https://singlecell.broadinstitute.org/single_cell (Bi et al.)	Renal Cell Carcinoma	28929	33711272	Number of patients: 8<br>Age: 60.5(57.75-63.5)<br>Sex: Male(6)/Female(2)<br>Stage: I(1)/IV(7)
https://www.ncbi.nlm.nih.gov/sra/PRJNA705464 (Krishna et al.)	Renal Cell Carcinoma	149159	33861994	Number of patients: 6<br>Age: 60(54.75-63.75)<br>Sex: Male(4)/Female(2)<br>Stage: III(2)/IV(4)

Table S4: List of pan-cancer transcriptomic datasets.

Dataset	Demographic information	Clinical information
TCGA-BLCA	Number of patients: 411<br>Age:69(60-76)<br>Sex: Male(303)/Female(108)<br>Race: Asian(44)/Black(23)/White(327)	OS status: Alive(226)/Dead(108)<br>OS time (day): 536(330-948)<br>AJCC Clinical M: M0(196)/M1(11)<br>AJCC Clinical N: N0(237)/N1(46)/N2(75)/N3(8)<br>AJCC Clinical T: T1(3)/T2(119)/T3(194)/T4(58)<br>AJCC Clinical Stage: StageI(2)/StageII(130)/StageIII(140)/StageIV(134)
TCGA-BRCA	Number of patients: 1104<br>Age:58(49-67)<br>Sex: Male(12)/Female(1091)<br>Race: Asian(61)/Black(183)/White(764)	OS status: Alive(928)/Dead(154)<br>OS time (day): 826.50(463.00-1685.25)<br>AJCC Clinical M: M0(901)/M1(22)<br>AJCC Clinical N: N0(514)/N1(360)/N2(120)/N3(76)<br>AJCC Clinical T: T1(279)/T2(631)/T3(137)/T4(40)<br>AJCC Clinical Stage: StageI(181)/StageII(619)/StageIII(247)/StageIV(20)
TCGA-COAD	Number of patients: 471<br>Age:69(58-77)<br>Sex: Male(246)/Female(223)<br>Race: Asian(11)/Black(62)/White(224)	OS status: Alive(325)/Dead(96)<br>OS time (day): 681(396-1094.25)<br>AJCC Clinical M: M0(333)/M1(64)<br>AJCC Clinical N: N0(267)/N1(105)/N2(82)

		AJCC Clinical T: T1(11)/T2(77)/T3(309)/T4(56)
		AJCC Clinical Stage: StageI(75)/StageII(176)/StageIII(128)/StageIV(64)
		OS status: Alive(357)/Dead(173)
		OS time (day): 1238.00(533.75-1928.75)
	Number of patients: 530	DFS status: Disease-free(110)/Recurred(17)
	Age:61(52-70)	DFS time (month): 38.63(19.91-72.92)
TCGA-KIRC	Sex: Male(344)/Female(186)	AJCC Clinical M: M0(420)/M1(78)
	Race: Asian(8)/Black(56)/White(459)	AJCC Clinical N: N0(239)/N1(16)
		AJCC Clinical T: T1(271)/T2(69)/T3(179)/T4(11)
		AJCC Clinical Stage: StageI(265)/StageII(57)/StageIII(123)/StageIV(82)
		OS status: Alive(237)/Dead(131)
	Number of patients: 374	OS time (day): 601(344.75-1102.25)
	Age:61(52-69)	AJCC Clinical M: M0(266)/M1(4)
TCGA-LIHC	Sex: Male(253)/Female(121)	AJCC Clinical N: N0(252)/N1(4)
	Race: Asian(160)/Black(17)/White(185)	AJCC Clinical T: T1(181)/T2(94)/T3(80)/T4(13)
		AJCC Clinical Stage: StageI(171)/StageII(86)/StageIII(85)/StageIV(5)
		OS status: Alive(328)/Dead(185)
	Number of patients: 374	OS time (day): 666(424-1130)
	Age:66(59-72)	AJCC Clinical M: M0(344)/M1(25)
TCGA-LUAD	Sex: Male(244)/Female(282)	AJCC Clinical N: N0(330)/N1(95)/N3(2)
	Race: Asian(7)/Black(54)/White(398)	AJCC Clinical T: T1(168)/T2(276)/T3(47)/T4(19)
		AJCC Clinical Stage: StageI(274)/StageII(121)/StageIII(84)/StageIV(26)
	Number of patients: 178	OS status: Alive(85)/Dead(92)
	Age:65(57-73)	OS time (day): 466(278-676)
TCGA-PAAD	Sex: Male(98)/Female(80)	AJCC Clinical M: M0(79)/M1(4)
	Race: Asian(11)/Black(6)/White(157)	AJCC Clinical N: N0(49)/N1(123)

		AJCC Clinical T: T1(7)/T2(24)/T3(141)/T4(3)<br>AJCC Clinical Stage: StageI(21)/StageII(146)/StageIII(3)/StageIV(4)
TCGA-READ	Number of patients: 167<br>Age:66(57-72)<br>Sex: Male(91)/Female(75)<br>Race: Asian(1)/Black(6)/White(81)	OS status: Alive(130)/Dead(28)<br>OS time (day): 638.5(400-1120.5)<br>AJCC Clinical M: M0(126)/M1(23)<br>AJCC Clinical N: N0(84)/N1(45)/N2(33)<br>AJCC Clinical T: T1(9)/T2(28)/T3(113)/T4(14)<br>AJCC Clinical Stage: StageI(30)/StageII(51)/StageIII(51)/StageIV(24)
TCGA-SKCM	Number of patients: 471<br>Age:58(48-71)<br>Sex: Male(292)/Female(179)<br>Race: Asian(12)/Black(1)/White(448)	OS status: Alive(235)/Dead(222)<br>OS time (day): 1124(505-2402)<br>AJCC Clinical M: M0(416)/M1(24)<br>AJCC Clinical N: N0(234)/N1(74)/N2(49)/N3(54)<br>AJCC Clinical T: T1(41)/T2(78)/T3(90)/T4(152)<br>AJCC Clinical Stage: StageI(76)/StageII(140)/StageIII(170)/StageIV(23)
RCC-Braun_2020	Number of patients: 311<br>Age:63(56-69)<br>Sex: Male(229)/Female(82)	OS status: Alive(80)/Dead(231)<br>OS time (month): 20.99(8.61-42.73)<br>PFS status: Progression-free(35)/Progression (276)<br>PFS time (month): 3.58(1.71-8.45)<br>Treatment status: CRPR(44)/PD(106)/SD(131)/NE(30)
JAVELIN-Renal-101	Number of patients: 726<br>Age:63(56-69)<br>Sex: Male(584)/Female(178)	PFS status: Progression-free(358)/Progression (368)<br>PFS time (month): 6.93(3.15-10.92)
